# Porcine Deltacoronavirus M Protein Binds NLRP3 to Promote Inflammasome Assembly via Competition with TRIM31

**DOI:** 10.1002/advs.76393

**Published:** 2026-06-30

**Authors:** Jinhui Hou, Fangfang Han, Anqi Liu, Yang Yu, Rui Zhao, Zhengting Shi, Yanrong Lv, Jinli Liu, Shaopo Zu, Zhanyong Wei, Jin Yuan, Hui Hu

**Affiliations:** ^1^ College of Veterinary Medicine Henan Agricultural University Zhengzhou P. R. China; ^2^ Ministry of Education Key Laboratory for Animal Pathogens and Biosafety Zhengzhou P. R. China; ^3^ Henan Province Key Laboratory for Animal Food Pathogens Surveillance Zhengzhou P. R. China

**Keywords:** M protein, NLRP3 inflammasome, porcine deltacoronavirus (PDCoV), TRIM31, ubiquitination

## Abstract

Porcine deltacoronavirus (PDCoV) infection induces severe intestinal inflammation and acute diarrhea in piglets, resulting substantial economic losses. The NLR family pyrin domain containing 3 (NLRP3) inflammasome, a key component of the innate immune system, drives interleukin (IL)‐1β maturation, and its excessive activation is closely linked to viral pathogenesis. However, its role in PDCoV infection remains unclear. Here, we report that PDCoV infection induces IL‐1β maturation through NLRP3 inflammasome activation both in *vivo* and in *vitro*, and the M protein promotes NLRP3 inflammasome assembly and activation. Mechanistically, M protein directly bound the LRR domain of the NLRP3 protein. Seven residues (M116, H201, T205, K207, R212, Y214, and M217) in the β‐sheet core of the M protein formed hydrogen bonds with eight residues (Y861, R920, E949, E1007, Y1009, K1015, N1011, and E1033) in the α‐helix groove of the LRR domain, serving as critical determinants of binding. Moreover, the M protein protects NLRP3 from proteasomal degradation by removing tripartite motif‐containing 31‐mediated K48‐linked polyubiquitin chains on NLRP3, thereby stabilizing NLRP3 protein. Collectively, these findings reveal a novel mechanism of NLRP3 inflammasome activation during PDCoV infection, offering valuable insights into PDCoV pathogenesis that may facilitate the design of antiviral agents against PDCoV.

## Graphical abstract

Porcine deltacoronavirus (PDCoV) infection induces severe intestinal inflammation and acute diarrhea in piglets, yet the molecular mechanism remains incompletely understood. The M protein activates NLRP3 inflammasome through dual mechanisms: direct binding to the NLRP3 LRR domain and disruption of TRIM31‐mediated K48‐linked ubiquitination. These findings establish a novel mechanism of M protein‐driven inflammasome hyperactivation.

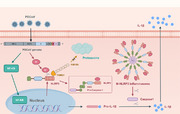

## 1 Introduction

Coronaviruses (CoVs; order *Nidovirales*, family *Coronaviridae*, and subfamily *Coronavirinae*) are enveloped, single‐stranded, positive‐sense RNA viruses with the largest genome (approximately 26–32 kb) among all known RNA viruses. CoVs belong to the order *Nidovirales*, family *Coronaviridae*, and subfamily *Coronavirinae*, and are classified into four genera: *Alpha‐coronavirus*, *Beta‐coronavirus*, *Gamma‐coronavirus*, and *Delta‐coronavirus* [1, 2]. CoVs encode essential structural proteins including spike (S), envelope (E), membrane (M), and nucleocapsid (N) proteins; several nonstructural proteins are responsible for viral replication and host immune evasion [3, 4]. These viruses primarily caused respiratory and gastrointestinal diseases in humans and animals. Porcine deltacoronavirus (PDCoV; genus Deltacoronavirus) has been shown to cause acute diarrhea, vomiting, dehydration and mortality in piglets [5]. It was first identified in Hong Kong, China in 2012, and subsequently spread rapidly across North America, Asia, and Europe [6, 7]. More importantly, PDCoV has been shown to have broad cellular tropism with the capability to infect a wide variety of cells from human and diverse animals, implicating its ability to hold high risks of cross‐species transmission [8, 9]. This cross‐species transmission potential of PDCoV has been verified in some experimental infections, with limited infection in chickens, calves, turkeys, and mice, highlighting its zoonotic potential [8, 9, 10, 11, 12]. In addition PDCoV has been isolated from three Haitian children with acute undifferentiated fever, providing the first evidence of potential zoonotic transmission in humans [13, 14]. However, the molecular mechanisms underlying PDCoV pathogenesis and interspecies transmission remain largely unknown.

PDCoV primarily infects epithelial cells in the small intestine of piglets, leading to thinning and hyalinization of the intestinal wall, villus atrophy, and intestinal inflammation [15, 16, 17, 18, 19, 20]. In *vitro* and in *vivo* models have shown that PDCoV infection upregulates pro‐inflammatory cytokines, including the tumor necrosis factor α (TNF‐α), interleukin‐1β (IL‐1β), IL‐6, IL‐8, with especially high expression of IL‐1β both in swine intestinal tissue and serum [21, 22, 23]. IL‐1β plays a critical role in various inflammatory diseases [24, 25], with recent studies showing associations to inflammatory bowel disease pathogenesis via disruption of intestinal epithelial tight junction barriers to promote inflammatory cell infiltration and mucosal damage [26]. However, the molecular mechanisms underlying PDCoV‐mediated IL‐1β expression remain poorly understood.

NLR family pyrin domain containing 3 (NLRP3) protein expression and inflammasome assembly lead to caspase‐1‐mediated maturation and release of IL‐1β, triggering inflammation and pyroptosis, which are critical for antiviral immunity and inflammation [27, 28, 29]. The NLRP3 inflammasome comprises the sensor protein NLRP3, the adaptor protein apoptosis‐associated speck‐like protein containing a CARD domain (ASC), and the effector protein caspase‐1 (cysteine‐aspartic acid protease 1). It senses viral pathogen‐associated molecular patterns to mediate innate immunity and IL‐1β maturation [30, 31, 32]. Canonical NLRP3 activation requires dual signals, as follows: nuclear factor (NF)‐κB priming (Signal 1) upregulates NLRP3 and pro‐IL‐1β expression, while inflammasome assembly signals (Signal 2) trigger NLRP3 inflammasome assembly, caspase‐1 cleavage, and IL‐1β maturation [33, 34, 35]. Multiple RNA viruses, including severe acute respiratory syndrome coronavirus (SARS‐CoV), severe acute respiratory syndrome coronavirus 2 (SARS‐CoV‐2), transmissible gastroenteritis virus (TGEV), porcine epidemic diarrhea virus (PEDV), porcine reproductive and respiratory syndrome virus (PRRSV), newcastle disease virus (NDV) and influenza A virus (IAV), have been shown to activate the NLRP3 inflammasome [18, 19, 20, 21, 22, 23]. However, there are important differences in the underlying mechanisms and pathological effects. For example, the N protein of SARS‐CoV‐2 has been demonstrated to directly bind to NLRP3, promoting NLRP3‐ASC oligomerization and thereby accelerating inflammasome assembly [36]. This leads to the maturation and secretion of IL‐1β and IL‐6, ultimately causing lung injury and acute respiratory distress syndrome (ARDS) [36, 37]. PEDV infection primarily induced mitochondrial dysfunction, mitochondrial DNA release, and reactive oxygen species (ROS) generation, and these factors serve as damage‐associated molecular patterns (DAMPs) that trigger inflammasome activation and upregulate pro‐IL‐1β expression via NF‐κB signaling [38]. Similarly, PRRSV‐mediated NLRP3 activation induces inflammatory cytokine secretion and pyroptosis in infected cells, releasing intracellular contents and exacerbating inflammation [39]. However, whether PDCoV infection activates the NLRP3 inflammasome and the associated mechanisms remain largely unknown.

Hence, this study aimed to investigate the role of PDCoV infection in NLRP3 inflammasome activation and IL‐1β maturation both in *vivo* and in *vitro*. We first demonstrated that PDCoV infection induces host inflammatory responses by activating the NLRP3 inflammasome, thereby promoting IL‐1β maturation and secretion. Furthermore, the PDCoV M protein acts as a direct regulator of NLRP3 inflammasome assembly by directly binding to the LRR domain of the NLRP3. Mechanistically, the PDCoV M protein competes with E3 ubiquitin ligase TRIM31 for NLRP3 binding, removing K48‐linked ubiquitination and thereby stabilizing NLRP3 to promote inflammasome activation. These findings reveal a novel mechanism by which the PDCoV M protein regulates the NLRP3 inflammasome and provide new insights into PDCoV‐mediated host inflammation.

## 2 Results

### 2.1 PDCoV Infection Induces Intestinal Inflammatory Responses in Piglets

**FIGURE 1:**
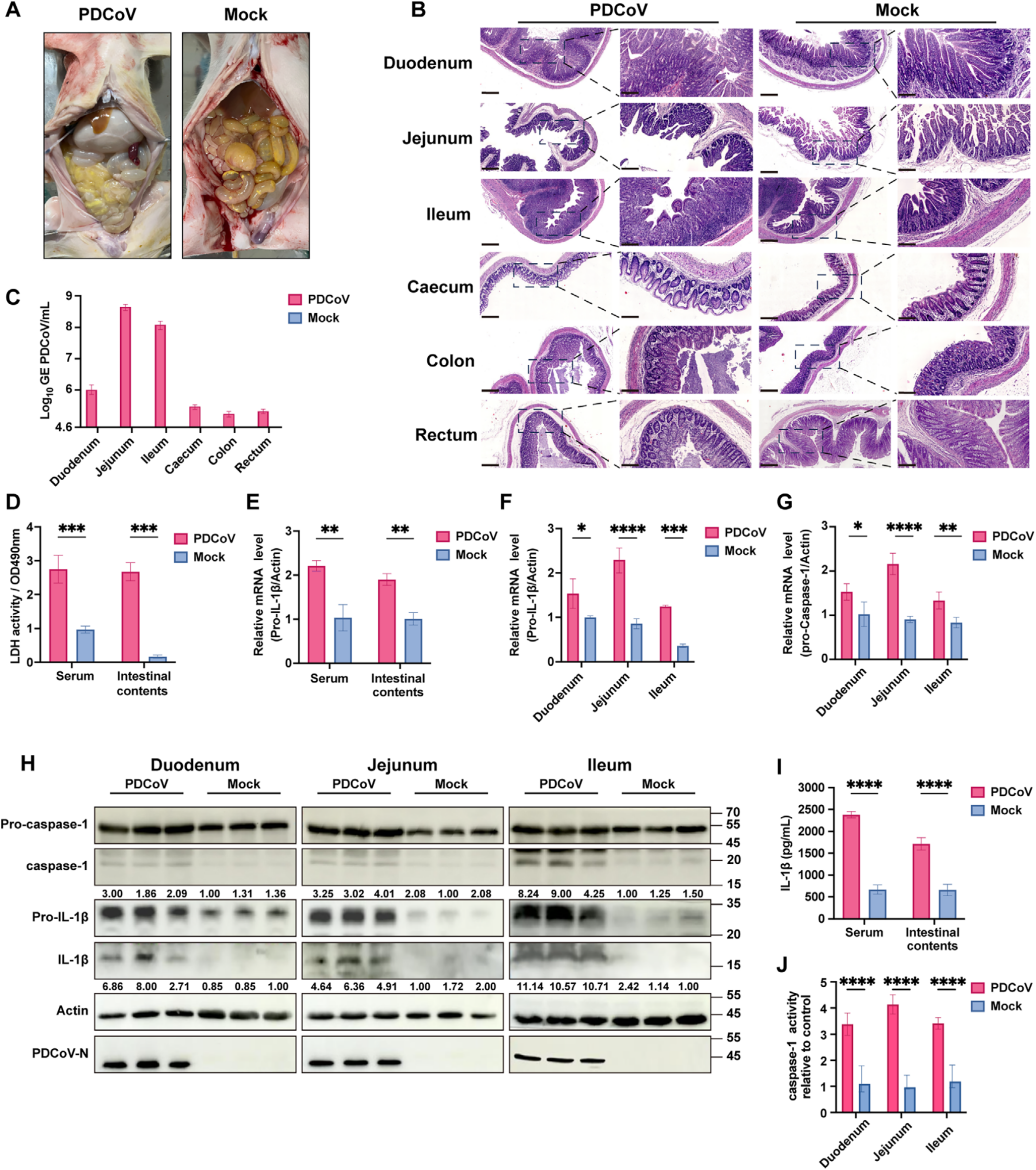
PDCoV infection induces inflammatory responses in *vivo*. (A) Gross intestinal lesions in piglets at 3 days post‐infection (dpi) with PDCoV. Representative images showing typical pathological changes in infected and control animals. (B) Hematoxylin and eosin (H&E) staining of intestinal tissues from PDCoV‐infected piglets at 3 dpi. Scale bars: 500 µm (low magnification) and 150 µm (high magnification). (C) PDCoV viral load quantification in intestines at 3 dpi determined by quantitative reverse transcription PCR (qRT‐PCR). (D) LDH release in serum and intestinal contents at 3 dpi measured by enzyme‐linked immunosorbent assay (ELISA), with absorbance detected at OD490 nm. (E) IL‐1β mRNA levels in serum and intestinal contents at 3 dpi determined by qRT‐PCR. (F,G) mRNA levels of IL‐1β (F) and pro‐caspase‐1 (G) in the small intestine at 3 dpi determined by qRT‐PCR. (H) Caspase‐1 cleavage and IL‐1β maturation in the small intestine at 3 dpi determined by western blot. Lanes 1–3: PDCoV‐infected piglets; lanes 4–6: mock‐infected control piglets. Representative results from three independent experiments are shown. (I,J) IL‐1β protein levels in serum and intestinal contents and caspase‐1 activity in the small intestinal tissues at 3 dpi measured by ELISA. Data are representative of three independent experiments. Data are expressed as mean ± SD, *n* = 3. Statistical significance was determined using one‐way ANOVA followed by Tukey's multiple comparisons test or two‐way ANOVA followed by Bonferroni's multiple comparisons test. *p < 0.05, **p < 0.01, ***p < 0.001, ****p < 0.0001.

To investigate the relationship between PDCoV infection and intestinal inflammation, we used the PDCoV‐infected piglet model established in our laboratory [6, 21]. Notably, 5‐day‐old piglets developed severe diarrhea at 3 days post‐infection (dpi) with the PDCoV HNZK‐02 strain. These piglets were subsequently euthanized, and their intestinal segments were harvested for histopathological analysis. Necropsy revealed thinned, translucent small intestinal walls in PDCoV‐infected piglets (Figure 1A), and histological examination of the jejunum and ileum showed epithelial desquamation, villous atrophy, and inflammatory cell infiltration (Figure 1B). Viral load analysis confirmed that PDCoV predominantly colonized the small intestine, with the highest viral titers in the jejunum and ileum (Figure 1C), which were the regions exhibiting the most severe tissue damage.

To further characterize the intestinal damage induced by PDCoV infection, we measured lactate dehydrogenase (LDH) release by enzyme‐linked immunosorbent assay (ELISA), which revealed elevated levels in both serum and intestinal contents post‐infection, indicating that PDCoV infection causes intestinal damage (Figure 1D). Next, to assess whether this pathology involved inflammasome‐mediated inflammation, the mRNA expression levels of key inflammatory mediators in piglet serum and intestinal contents were evaluated. Critical factors, including IL‐1β, IL‐6, IL‐8, and TNF‐α, were significantly upregulated after PDCoV infection (Figure 1E and Figure S1A–G). Furthermore, the upstream mechanism driving pro‐inflammatory cytokine expression was explored by examining NF‐κB signaling. Western blot analysis revealed markedly increased phosphorylated p65 (p‐p65) levels in the small intestinal tissues of infected piglets, indicating that PDCoV infection activates NF‐κB signaling, which likely promotes the transcriptional upregulation of these cytokines (Figure S1H).

Among these mediators, IL‐1β is a pivotal pro‐inflammatory cytokine that drives inflammatory damage. It is produced via inflammasome‐mediated cleavage of pro‐IL‐1β by mature caspase‐1, which is mediated by the inflammasome [40, 41, 42]. Therefore, we quantified IL‐1β levels in both systemic circulation and local intestinal environments. Quantitative reverse transcription polymerase chain reaction (qRT‐PCR) showed that PDCoV infection significantly upregulated IL‐1β mRNA expression in the serum, intestinal contents, and small intestinal tissues (duodenum, jejunum and ileum) (Figure 1E,F and Table 1). We next examined the precursor and maturation of IL‐1β by western blot. A clear increase was observed in both pro‐IL‐1β (p31) and cleaved IL‐1β (p17) levels in the small intestinal tissues after PDCoV infection (Figure 1H). ELISA results further confirmed that PDCoV infection significantly increased IL‐1β secretion in the serum and intestinal contents, as well as caspase‐1 activity in the small intestinal tissues (Figure 1I,J). Concurrently, we assessed caspase‐1 mRNA expression and protein activation in small intestinal tissues using qRT‐PCR and western blot. As expected, PDCoV infection increased caspase‐1 mRNA, pro‐caspase‐1 protein, and cleaved caspase‐1 (activated caspase‐1, p20) levels (Figure 1G,H and Table 1). Collectively, these findings demonstrate that PDCoV infection triggers intestinal inflammatory responses via caspase‐1 activation and subsequent IL‐1β maturation in piglet intestines.

**TABLE 1:** Primers for qRT‐PCR to detect NLRP3 inflammasome mRNA levels.

Primers	Genbank	Primer sequences (5′→3′)	Length/bp
β‐actin	ON164673.1	F:TTCCAGCCCTCCTTCCTG R:AGGTCCTTGCGGATGTCG	94
PDCoV‐S115	MH708123	F:CGTTAACCTCTTCTCACCACTT R:GCTGAGAGTCTGGTTGGTTATT	115
p IL‐1β	NM_214055.1	F:GTCTGTCATCGTGGCAGTGGA R:GGAGGGATTCTTCATCGGCTT	43
p Caspase‐1	NM_214162.1	F:GCCTTGCCCTCATAATCT R:ACATCTGGGACTTCTTCG	277
p ASC	XM_003124468.5	F:TCGTGGACCAGCATCG R::TGAAGAGCCTCCTCATTTT	150
p NLRP3	NM_001256770.2	F:ATTCACTGTCGGGAGGT R:CTATGGGTGGGTTTGG	88
p IL‐6	NM_214399.1	F:GACCCTGAGGCAAAAGGGAA R:TCGTTCTGTGACTGCAGCTT	96
p IL‐8	NM_213867.1	F:TGGCAGTTTTCCTGCTTTCT R:CAGTGGGGTCCACTCTCAAT	154
p TNF‐α	NM_214022.1	F:GCCCAAGGACTCAGATCATC R:GGCATTGGCATACCCACTCT	111
p IFN‐a	NM_001164843.1	F:GCTCCTGGCACAAATGAGGA R:ATGGCTTGAGCCTTCTGGAC	114
p CCL2	NM_214214.1	F:ATCTTCAAGACCATCGCGGG R:TCAAGGCTTCGGAGTTTGGTT	108
p CXCL2	NM_001444936.1	F:GACCGTGCAAGGAATTCACC R::TGCGGGGTTGAGACAAACTT	127
p CCL20	XM_005672261.3	F:ATCATGGGCTTCACACAGCA R:TCTGTGGATCTGCACACACG	100

### 2.2 PDCoV Infection Induces IL‐1β Maturation and Secretion In Vitro

**FIGURE 2:**
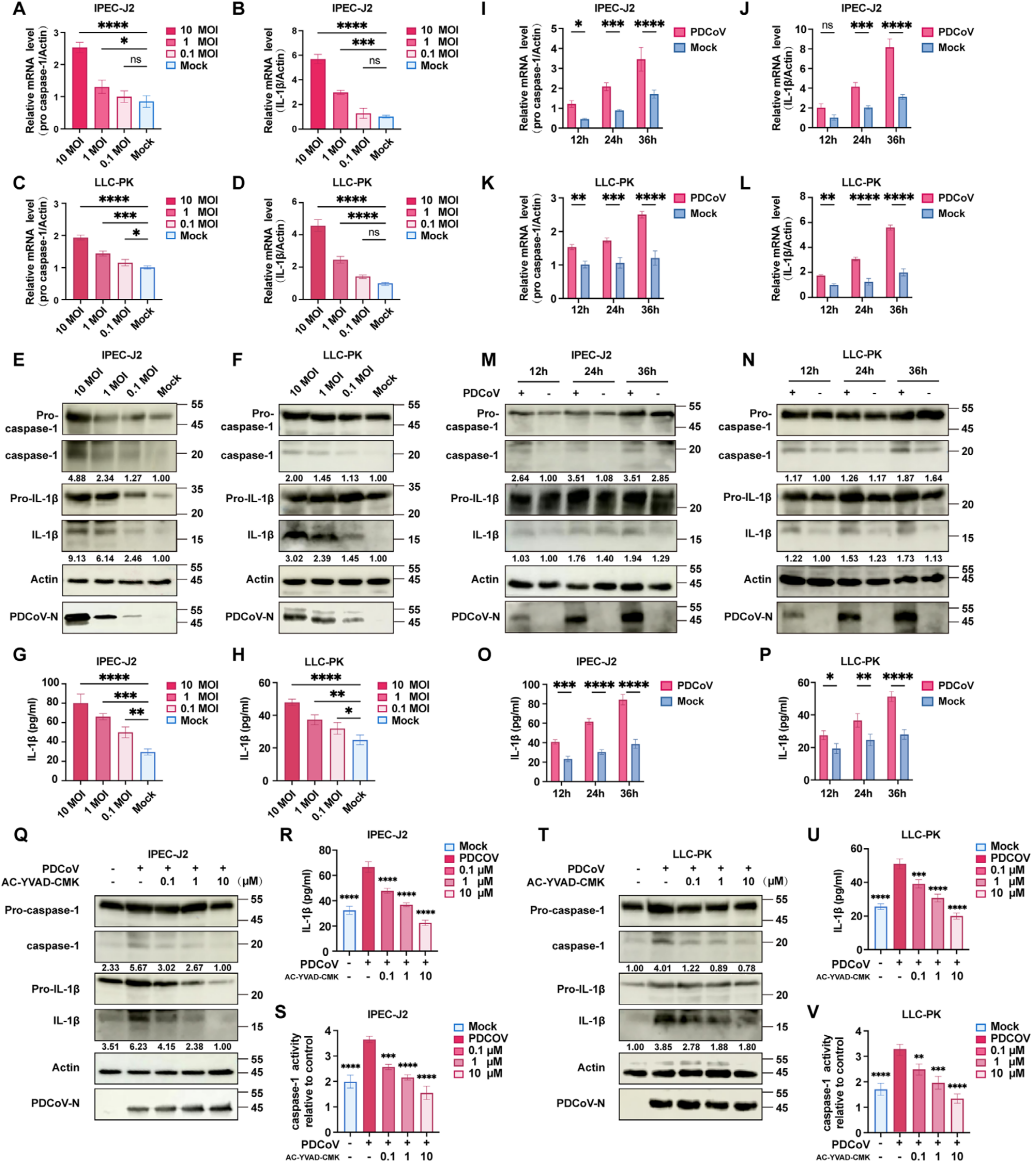
PDCoV infection induces IL‐1β maturation and secretion in *vitro*. (A‐D) mRNA levels of caspase‐1 (A, C) and IL‐1β (B,D) in IPEC‐J2 and LLC‐PK cells infected with PDCoV at different MOIs (10, 1, and 0.1) for 24 h determined by qRT‐PCR. (E‐F) Caspase‐1 cleavage and IL‐1β maturation in IPEC‐J2 (E) and LLC‐PK (F) cells infected with PDCoV at different MOIs (10, 1, and 0.1) for 24 h determined by western blot. (G,H) IL‐1β secretion levels in culture supernatants of IPEC‐J2 (G) and LLC‐PK (H) cells infected with PDCoV at different MOIs (10, 1, and 0.1) for 24 h measured by ELISA. (I–L) mRNA levels of caspase‐1 (I, K) and IL‐1β (J, L) in IPEC‐J2 and LLC‐PK cells infected with PDCoV (MOI = 1) at different time points (12, 24, and 36 h) determined by qRT‐PCR. (M‐N) Caspase‐1 cleavage and IL‐1β maturation in IPEC‐J2 (M) and LLC‐PK (N) cells infected with PDCoV (MOI = 1) at different time points (12, 24, and 36 h) determined by western blot. (O,P) IL‐1β secretion levels in culture supernatants of IPEC‐J2 (O) and LLC‐PK (P) cells infected with PDCoV (MOI = 1) at different time points (12, 24, and 36 h) measured by ELISA. (Q–V) Effect of caspase‐1 inhibitor Ac‐YVAD‐cmk on PDCoV‐induced inflammasome activation. IPEC‐J2 and LLC‐PK cells treated with Ac‐YVAD‐cmk at different concentrations (0.1, 1, and 10 µM) for 3 h, then infected with PDCoV at MOI = 1 for 36 h. Caspase‐1 cleavage and IL‐1β maturation determined by western blot (Q, T). IL‐1β secretion levels measured by ELISA (R, U). Caspase‐1 enzyme activity measured by ELISA (S, V). Representative results from three independent experiments are shown. Data are expressed as mean ± SD, *n* = 3. Statistical significance was determined using one‐way ANOVA followed by Tukey's multiple comparisons test or two‐way ANOVA followed by Bonferroni's multiple comparisons test. *p < 0.05, **p < 0.01, ***p < 0.001, ****p < 0.0001.

To further elucidate the PDCoV‐induced inflammasome activation observed in *vivo*, we established PDCoV‐infection cell models using porcine intestinal epithelial (IPEC‐J2) and LLC porcine kidney (LLC‐PK) cells, the primary target cells for PDCoV infection in piglets and the most suitable cell line for virus replication, respectively [43, 44, 45]. IPEC‐J2 and LLC‐PK cells were infected with PDCoV at multiplicities of infection (MOIs) of 10, 1, and 0.1, and samples were harvested at 24 h post‐infection (hpi). The mRNA and protein expression of caspase‐1 and IL‐1β were analyzed by qRT‐PCR and western blot. These assays revealed dose‐dependent increases in the mRNA and protein levels of IL‐1β and caspase‐1 during PDCoV infection (Figure 2A–F). Consistently, ELISA confirmed elevated secretion of IL‐1β in supernatants and caspase‐1 activity in cell lysates (Figure 2G,H and Figure S2E,F). To further substantiate inflammasome activation, we examined ASC oligomerization and LDH release. Western blot analysis demonstrated a significant dose‐dependent increase in ASC oligomerization in both IPEC‐J2 and LLC‐PK cells after PDCoV infection (Figure S2A,B). LDH release into supernatants also increased in a dose‐dependent manner (Figure S2I,J), aligning with enhanced extracellular secretion of IL‐1β observed under the same conditions.

Subsequently, IPEC‐J2 and LLC‐PK cells were infected with PDCoV (MOI = 1), and samples were harvested at 12, 24, and 36 hpi. IL‐1β and caspase‐1 showed progressive upregulation at both the mRNA and protein levels, correlating with PDCoV infection duration (Figure 2I–P and Figure S2G,H). Consistently, ASC oligomerization and LDH release increased in a time‐dependent manner in both cell lines, further corroborating PDCoV‐induced temporal dynamics of inflammasome activation (Figure S2C,D and K,L). Collectively, these findings demonstrate that PDCoV infection promotes caspase‐1 cleavage, IL‐1β maturation, ASC oligomerization, and LDH release, which serve as hallmarks of inflammasome activation, consistent with our in *vivo* findings.

To determine whether PDCoV‐induced IL‐1β secretion depends on caspase‐1, we pretreated IPEC‐J2 and LLC‐PK cells with the caspase‐1 specific inhibitor Ac‐YVAD‐cmk (0.1, 1, or 10 µM) for 3 h, followed by PDCoV infection (MOI = 1) for 36 h. Western blot analysis showed that caspase‐1 cleavage inhibition reduced IL‐1β maturation (Figure 2Q,T). ELISA confirmed Ac‐YVAD‐cmk inhibited caspase‐1 activity and IL‐1β secretion after PDCoV infection in a concentration‐dependent manner (Figure 2R,S and U,V). Consistently, LDH release into cell supernatants was also reduced in a concentration‐dependently manner following Ac‐YVAD‐cmk treatment in both IPEC‐J2 and LLC‐PK cells (Figure S2M,N). These results indicate that PDCoV‐induced IL‐1β maturation and secretion are dependent on caspase‐1 activation.

### 2.3 PDCoV Induces NLRP3 Inflammasome Activation

Previous studies have shown that the NLRP3 inflammasome regulates IL‑1β maturation and activation during infection with several RNA viruses, and that cellular NLRP3 protein levels are crucial for inflammasome activation and assembly [36, 46, 47, 48, 49, 50, 51, 52, 53]. We therefore investigated whether the inflammasome activation and assembly in PDCoV‐infected piglets.

**FIGURE 3:**
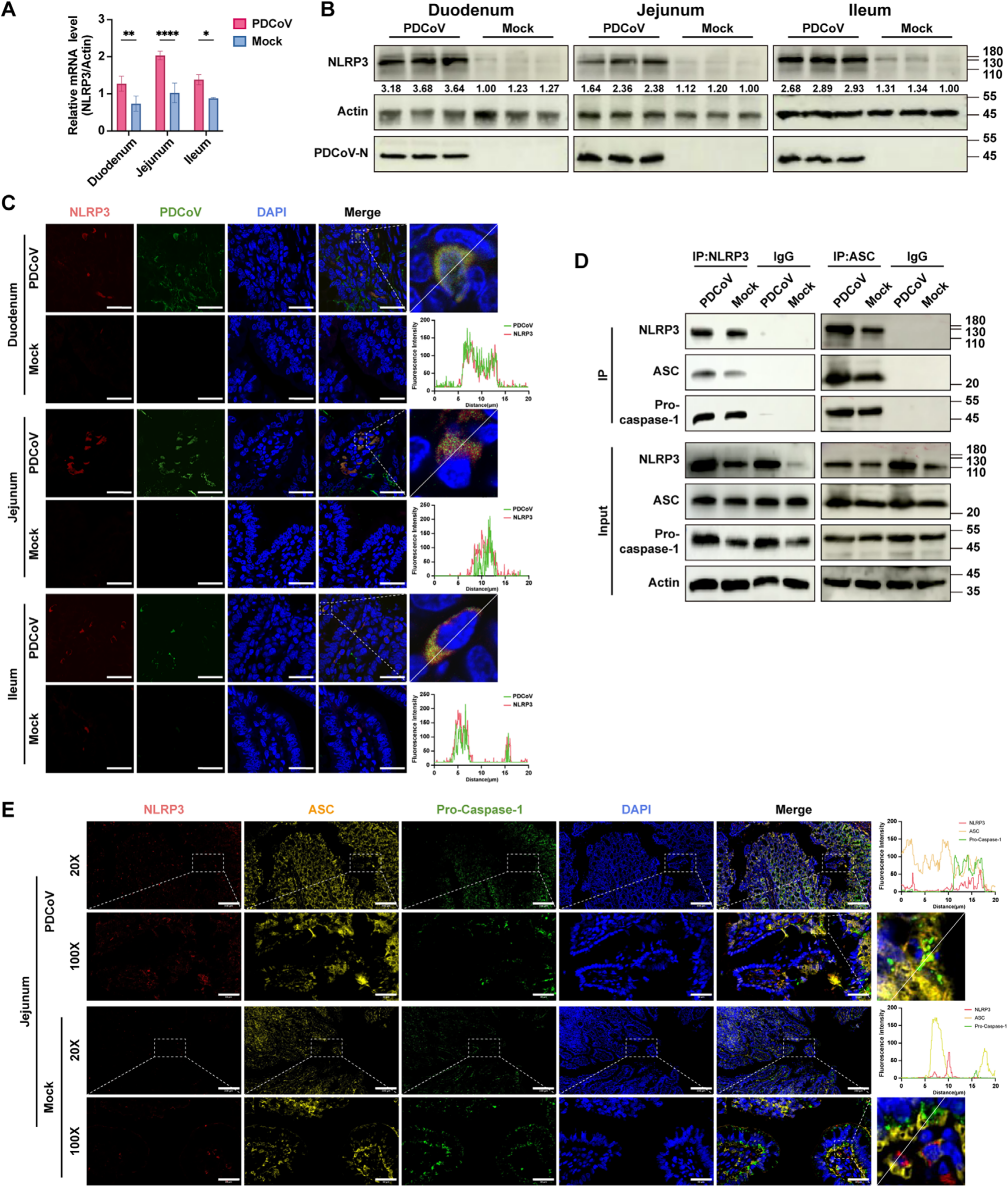
PDCoV induces NLRP3 inflammasome activation in *vivo*. (A) NLRP3 mRNA levels in the small intestine at 3 dpi determined by qRT‐PCR. (B) NLRP3 protein levels in the small intestine at 3 dpi determined by western blot. Lanes 1–3: PDCoV‐infected piglets; lanes 4–6: mock‐infected control piglets. (C) Co‐localization of PDCoV and NLRP3 proteins in small intestine visualized by confocal laser scanning microscopy. PDCoV (green), NLRP3 (red), nuclei (blue, DAPI). Scale bar: 30 µm. (D) PDCoV‐induced NLRP3 inflammasome assembly in jejunal tissues at 3 dpi detected by co‐immunoprecipitation (co‐IP). Tissue lysates were immunoprecipitated using anti‐NLRP3, anti‐ASC and control IgG antibodies and analyzed using anti‐ASC and anti‐pro‐caspase‐1 antibodies. (E) NLRP3 inflammasome assembly in jejunal tissues visualized by tyramide signal amplification (TSA). NLRP3 (red), ASC (yellow), pro‐caspase‐1 (green). Scale bars: 300 µm (overview) or 30 µm (high magnification). Representative results from three independent experiments are shown. Data are expressed as mean ± SD, *n* = 3. Statistical significance was determined using one‐way ANOVA followed by Tukey's multiple comparisons test or two‐way ANOVA followed by Bonferroni's multiple comparisons test. *p < 0.05, **p < 0.01, ***p < 0.001, ****p < 0.0001.

First, we assessed NLRP3 mRNA and protein levels in small intestinal tissues from PDCoV‐infected piglets. Notably, qRT‐PCR and western blot analyses revealed that PDCoV infection upregulated NLRP3 expression at both the mRNA and protein levels (Figure 3A,B and Table 1). Confocal immunofluorescence assays further confirmed that NLRP3 protein expression was significantly upregulated after PDCoV infection, and clear colocalization between PDCoV and NLRP3 was observed in swine small intestinal mucosal epithelial tissues (Figure 3C).

Next, we examined whether PDCoV infection promoted the assembly of the NLRP3 inflammasome complex. Co‐immunoprecipitation (co‐IP) assays showed significantly enhanced physical interactions among NLRP3, ASC, and pro‐caspase‐1 in the jejunal tissues from the PDCoV‐infected piglets compared with the mock group (Figure 3D). Correspondingly, tyramide signal amplification (TSA) analysis revealed markedly increased colocalization of NLRP3, ASC, and pro‐caspase‐1 in jejunal samples from infected piglets (Figure 3E). These findings indicate that PDCoV infection promotes the activation and assembly of the NLRP3 inflammasome in *vivo*.

**FIGURE 4:**
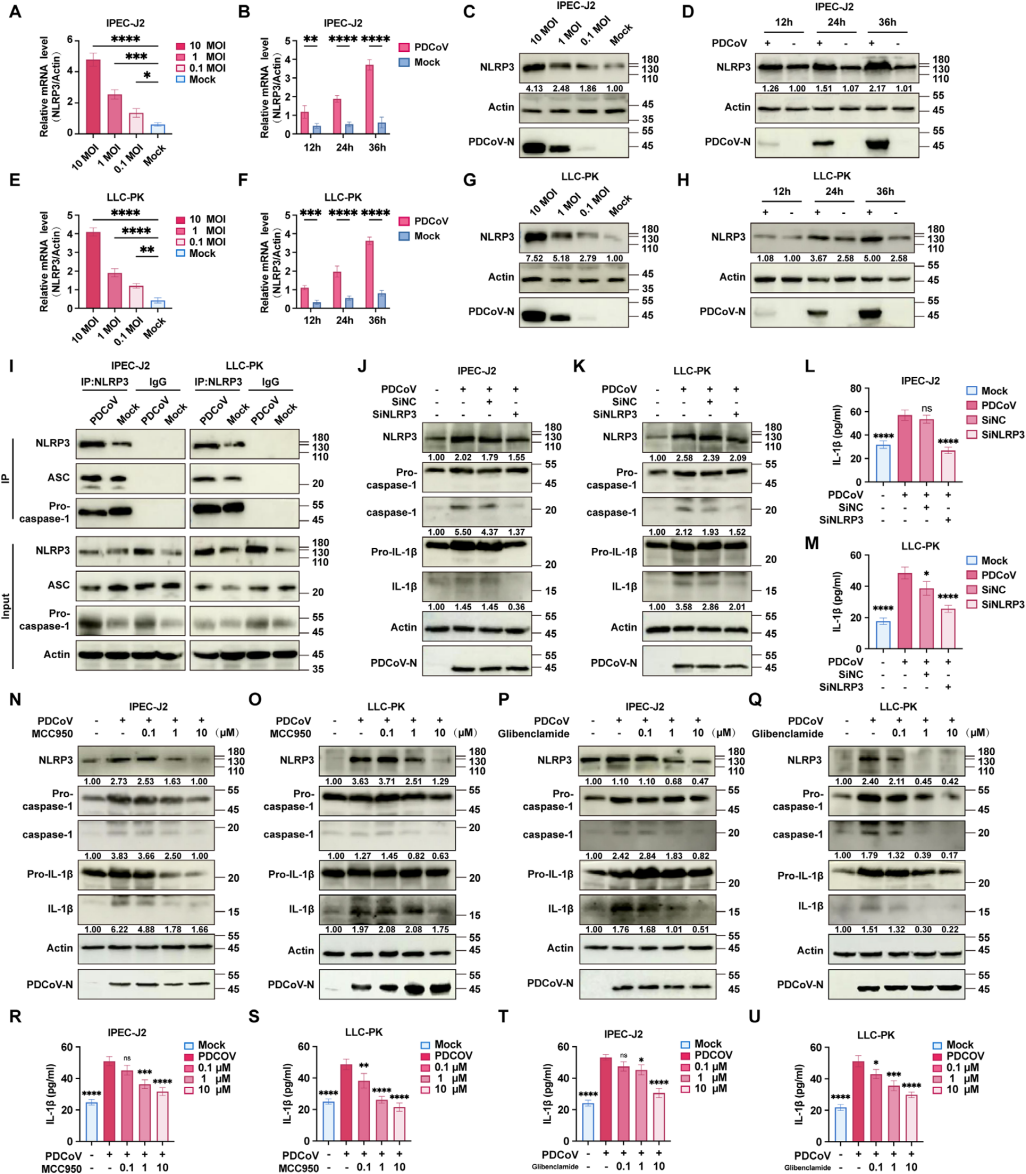
PDCoV infection induces IL‐1β maturation and secretion through the activation of NLRP3 inflammasomes. (A–H) NLRP3 expression analysis in PDCoV‐infected cells. IPEC‐J2 and LLC‐PK cells were infected with PDCoV at different MOIs (10, 1, 0.1) for 36 h and at MOI = 1 for different time points (12, 24, 36 h). NLRP3 mRNA determined by qRT‐PCR (A‐B, E‐F). NLRP3 protein levels determined by western blot (C‐D, G‐H). (I) PDCoV‐induced NLRP3 inflammasome assembly in IPEC‐J2 and LLC‐PK cells detected by co‐IP. Cells were infected with PDCoV (MOI = 1) for 36 h or mock‐infected. Cell lysates were immunoprecipitated using anti‐NLRP3 antibody or control IgG, then analyzed using anti‐NLRP3, anti‐ASC and anti‐pro‐caspase‐1 antibodies. (J–M) Effect of NLRP3 knockdown on PDCoV‐induced inflammasome activation. IPEC‐J2 and LLC‐PK cells were transfected with siNLRP3, or siNC (negative control) at 150 nM for 24 h, then infected with PDCoV (MOI = 1) for 36 h. NLRP3 inflammasome components expression levels determined by western blot (J‐K). IL‐1β secretion levels in culture supernatants in cell lysates of IPEC‐J2 and LLC‐PK cells measured by ELISA (L‐M). (N‐U) Effect of NLRP3 inflammasome inhibitors on PDCoV‐induced inflammasome activation. IPEC‐J2 and LLC‐PK cells were pretreated with MCC950 (N, O, R, S) or glibenclamide (P, Q, T, U) at different concentrations (0.1, 1, 10 µM) for 3 h, then infected with PDCoV (MOI = 1) for 36 h. NLRP3 inflammasome component expression levels determined by western blot (N‐Q). IL‐1β secretion levels measured by ELISA (R‐U). Representative results from three independent experiments are shown. Data are expressed as mean ± SD, *n* = 3. Statistical significance was determined using one‐way ANOVA followed by Tukey's multiple comparisons test or two‐way ANOVA followed by Bonferroni's multiple comparisons test. *p < 0.05, **p < 0.01, ***p < 0.001, ****p < 0.0001.

To validate whether similar phenomenon was observed in *vitro*, we evaluated NLRP3 inflammasome activation in IPEC‐J2 and LLC‐PK cells during PDCoV infection [6, 17, 43]. The results showed that PDCoV infection induced time‐ and dose‐dependent increases in NLRP3 mRNA and protein expression in both cell lines (Figure 4A–H and Table 1). Co‐IP assays showed that PDCoV infection enhanced the NLRP3, ASC, and pro‐caspase‐1 interactions in both cell lines (Figure 4I), and promoted the assembly of the NLRP3 inflammasome complex, demonstrating strengthened associations among ASC, NLRP3, and pro‐caspase‐1 under the same conditions (Figure S3A), thereby recapitulating the NLRP3 inflammasome assembly observed in *vitro*.

Next, we performed knockdown experiments using siRNA targeting NLRP3 (siNLRP3) to investigate the functional necessity of NLRP3, with non‐targeting siRNA (siNC) as a control. Following NLRP3 gene silencing, NLRP3 protein expression levels were markedly reduced; concomitantly, caspase‐1 cleavage and IL‐1β maturation were suppressed (Figure 4J,K). ELISA analysis showed that the levels of IL‐1β secretion in the supernatants were substantially reduced in siNLRP3‐transfected cells (Figure 4L,M). Consistently, LDH release into cell supernatants was also markedly diminished upon NLRP3 knockdown in both IPEC‐J2 and LLC‐PK cells (Figure S3B,C). Pharmacological validation using mechanistically distinct NLRP3 inhibitors, MCC950 (a specific NLRP3 ATPase inhibitor) and glibenclamide (a K^+^ channel blocker preventing NLRP3 activation), further confirmed these findings, showing dose‐dependently suppression of caspase‐1 cleavage and IL‐1β maturation and secretion by western blot and ELISA (Figure 4N–U). Correspondingly, LDH release was similarly attenuated in concentration‐dependent manner by both MCC950 and glibenclamide in both cell lines (Figure S3D–G). Altogether, these results demonstrate that PDCoV infection promotes IL‐1β maturation and secretion through NLRP3 inflammasome activation both in *vivo* and in *vitro*.

### 2.4 PDCoV M Protein Activates the NLRP3 Inflammasome

**FIGURE 5:**
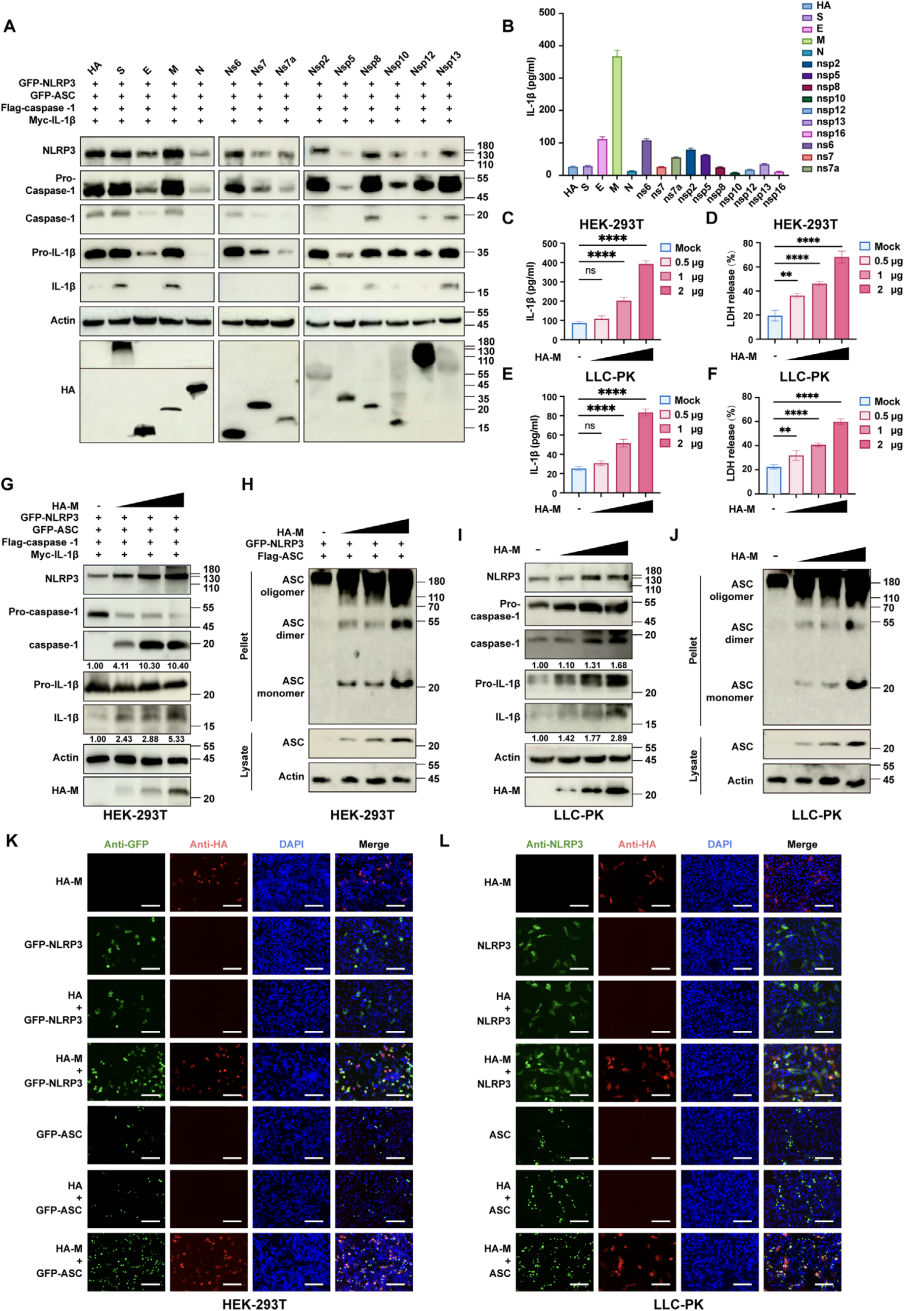
PDCoV M protein facilitates NLRP3 inflammasome activation. (A,B) Screening of PDCoV proteins on NLRP3 inflammasome activation. HEK‐293T cells were transfected with PDCoV proteins (HA‐tagged) along with NLRP3 inflammasome components (Myc‐pro‐IL‐1β, Flag‐pro‐caspase‐1, GFP‐ASC, GFP‐NLRP3) for 24 h. Empty vector (pCAGGS‐HA) served as the negative control. NLRP3 inflammasome components expression levels in cell lysates determined by western blot (A). IL‐1β secretion levels in culture supernatants measured by ELISA (B). (C–J) Dose‐dependent effects of PDCoV M protein on the NLRP3 inflammasome. HEK‐293T cells were co‐transfected with inflammasome components (GFP‐NLRP3, GFP‐ASC, Flag‐caspase‐1, Myc‐IL‐1β) and increasing amounts of HA‐M plasmids (C‐D, G‐H), and LLC‐PK cells only were transfected with increasing amounts of HA‐M plasmids (E‐F, I‐J). IL‐1β secretion levels (C, E) and LDH release (D, F) in culture supernatants of HEK‐293T and LLC‐PK measured by ELISA. NLRP3 inflammasome components expression levels in HEK‐293T and LLC‐PK determined by western blot (G, I). ASC oligomerization in the pellets and the total ASC in lysates as the input were determined by western blotting (H, J). (K,L) Effect of the M protein on ASC speck and NLRP3 spot formation. ASC speck and NLRP3 spot formation in HEK‐293T (K) and LLC‐PK (L) cells analyzed by immunofluorescence assay (IFA). HEK‐293T cells were co‐transfected with GFP‐NLRP3, or GFP‐ASC and HA‐M for 24 h, LLC‐PK cells were transfected with HA‐M for 24 h. NLRP3 and ASC (Green), PDCoV‐M (red). Scale bar: 150 µm. Representative results from three independent experiments are shown. Data are expressed as mean ± SD, *n* = 3. Statistical significance was determined using one‐way ANOVA followed by Tukey's multiple comparisons test or two‐way ANOVA followed by Bonferroni's multiple comparisons test. *p < 0.05, **p < 0.01, ***p < 0.001, ****p < 0.0001.

To identify the viral components that promote NLRP3 inflammasome activation, we reconstituted the inflammasome system in HEK‐293T cells by co‐expressing porcine NLRP3, ASC, pro‐caspase‐1, and pro‐IL‐1β (Table 2), and then co‐transfected with PDCoV viral proteins, respectively. Systematic screening of 13 PDCoV viral proteins revealed that PDCoV M protein was the most potent inducer of IL‐1β maturation and secretion (Figure 5A,B). Next, we assessed whether the M protein activates the NLRP3 inflammasome. HEK‐293T cells were transfected with different amounts of M protein expression plasmid (0.5, 1.0, or 2.0 µg) together with NLRP3 inflammasome component plasmids (0.5 µg). Western blot analysis revealed that the M protein enhanced caspase‐1 cleavage and IL‐1β maturation in a dose‐dependent manner (Figure 5G). To further confirm inflammasome activation, we examined ASC oligomerization in HEK‐293T cells co‐transfected with GFP‐NLRP3 and FLAG‐ASC constructs and increasing amounts of the M protein plasmid. Western blot analysis confirmed that ASC oligomerization increased in a dose‐dependent manner (Figure 5H), indicating inflammasome assembly. ELISA analysis confirmed that IL‐1β secretion and LDH release increased in a dose‐dependent manner (Figure 5C,D). Transfection of increasing amounts of the M protein expression plasmid into LLC‐PK cells alone was sufficiently activated the endogenous NLRP3 inflammasome and induced ASC oligomerization in a dose‐dependent manner, as shown by western blot (Figure 5I,J). Consistent with these findings, ELISA analysis showed that IL‐1β secretion and LDH release were upregulated in a dose‐dependent manner (Figure 5E,F). Subsequently, immunofluorescence assay (IFA) showed that the M protein promoted NLRP3 spot and ASC speck formation, which are hallmarks of inflammasome assembly, in both HEK‐293T and LLC‐PK cells (Figure 5K,L). Collectively, these results demonstrate that the PDCoV M protein activates the NLRP3 inflammasome.

**TABLE 2:** primers used for the construction of the NLRP3 inflammasome protein and its truncator.

Primers	Genbank	Primer sequences (5′→3′)	Length/bp
Myc‐IL‐1β	NM_214055.1	F:GTCTCATCATTTTGGCAAAGAATTCATGGCCATAGTACCTGAACC R:AAAGATCTGCTAGCTCGAGTTACAGGTCCTCCTCTGAGATAGCT TCTGCTCGGGAGAGAGGACT	798
Flag‐Caspase‐1	NM_214162.1	F:CTCATCATTTTTGGCAAAGAATTCATGGCCGATAAGGTGCTGAA R:AAAGATCTGCTAGCTCGAGTTACTTGTCATCGTCCGTTAGTAGT CATGTCCTGGGAAG	1215
GFP‐ASC	AB873106.1	F:TAGCGCTACCGGACTCAGATCTCGAGATGGGGTGCACGCGTGAC R:GCGGTACCGTCGACTGCAGAATTCCGCTCTGCTCCAGGTCGGC	591
GFP‐NLRP3	NM_001256770.2	F:CTACCGGACTCAGATCTCGAGATGAGCATGGCAAGCGTCC R:GTACCGTCGACTGCAGAATTCCCTACTGGGAAGGCTCAAA	3802
Flag‐NLRP3‐D1	NM_001256770.2	F:CTCATCATTTTGGCAAAGAATTCATGAGCATGGCAAGC R:AAAAGATCTGCTAGCTCGAGTTACTTGTCATCGTCGTCCTTGTAGT CCACAGGCTCAGAG	648
Flag‐NLRP3‐D2	NM_001256770.2	F:CTCATCATTTTGGCAAAGAATTCATGAGCATGGCAAGC R:AAAAGATCTGCTAGCTCGAGTTACTTGTCATCGTCGTCCTTGTAGTC AATCAGATACCCC	1716
Flag‐NLRP3‐D3	NM_001256770.2	F:CTCATCATTTTGGCAAAGAATTCATGTGGGACAATGCAAAT R:AAAGATCTGCTAGCTCGAGTTACTTGTCATCGTCGTCCTTGTA GTCCTGGGAAGGCTCAA	2832
Flag‐NLRP3‐D4	NM_001256770.2	F:TCATCATTTTGGCAAAGAATTCATGTTTGTTGTCCGTTTCC R:AAAGATCTGCTAGCTCGAGTTACTTGTCATCGTCGTCCTTGTA GTCCTGGGAAGGCTCAA	1395
Flag‐NLRP3‐D5	NM_001256770.2	F:CTCATCATTTTGGCAAAGAATTCATGCACACGGTAGTATTC R:AAAGATCTGCTAGCTCGAGTTACTTGTCATCGTCGTCCTTGTAGT CATTCCAGTTATTGC	1509
Myc‐M	AXP32218.1	F: TTATGGCCATGGAGGCCCGAATTCAGTCTGACGACGCAGAAGA R: TCCCCGCGGCCGCGGTACCTCGAGTTACATATACTTATACAG	653

### 2.5 PDCoV M Protein Interacts with NLRP3 through Binding to the LRR Domain

**FIGURE 6:**
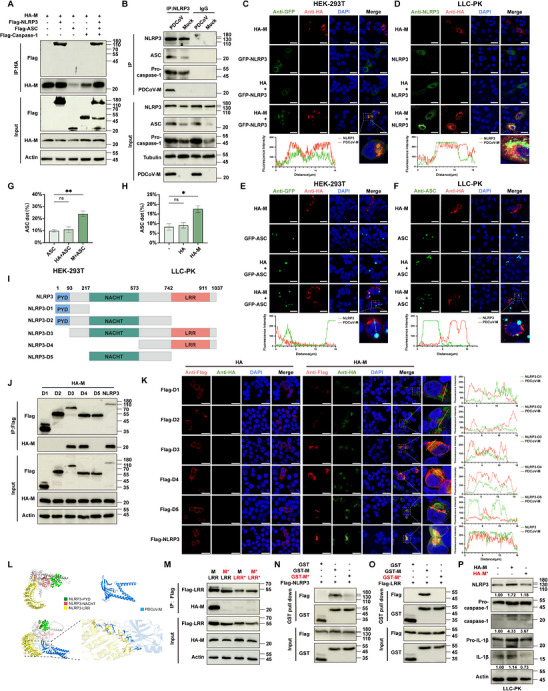
PDCoV M promotes NLRP3 inflammasome activation through interaction with LRR domain of NLRP3 protein. (A,B) PDCoV M protein interacts with NLRP3 inflammasome components. HEK‐293T cells were co‐transfected with HA‐M and Flag‐NLRP3, Flag‐ASC or Flag‐caspase‐1 for 24 h. Cell lysates were immunoprecipitated using anti‐HA antibody and analyzed using anti‐Flag, and anti‐HA antibodies (A). LLC‐PK cells were infected with PDCoV (MOI = 1) for 36 h. Cell lysates were immunoprecipitated using anti‐NLRP3 antibody or control IgG and analyzed using anti‐NLRP3, anti‐ASC, anti‐pro‐caspase‐1, and anti‐PDCoV M monoclonal antibodies (B). (C–F) Co‐localization analysis of PDCoV M protein with NLRP3 and ASC. Co‐localization of PDCoV‐M and NLRP3 spot (C,D) and ASC speck formation (E,F) in HEK‐293T and LLC‐PK cells visualized by confocal microscopy. HEK‐293T cells were co‐transfected with GFP‐NLRP3 or GFP‐ASC and HA‐M for 24 h. LLC‐PK cells were transfected with HA‐M for 24 h and stained for endogenous NLRP3 and ASC. Scale bar: 25 µm. (G,H) Quantification of ASC speck formation in HEK‐293T and LLC‐PK cells. Percentages of cells with ASC specks from experiments shown in panels E and F. (I) Schematic diagram of NLRP3 protein functional domains and truncated mutants. (J) PDCoV M protein interacts with NLRP3 protein functional domains. HEK‐293T cells were co‐transfected with HA‐M and Flag‐tagged NLRP3 domain (Flag‐D1, Flag‐D2, Flag‐D3, Flag‐D4, Flag‐D5) or full‐length Flag‐NLRP3 for 24 h. Cell lysates were immunoprecipitated using anti‐Flag antibody and analyzed using anti‐Flag, and anti‐HA antibodies. (K) Co‐localization of PDCoV M protein with NLRP3 protein functional domains. HEK‐293T cells were co‐transfected with Flag‐tagged NLRP3 domain (Flag‐D1, Flag‐D2, Flag‐D3, Flag‐D4, Flag‐D5) or full‐length Flag‐NLRP3 for 24 h, then analyzed by confocal microscopy. Scale bar: 25 µm. (L) Structural prediction of PDCoV M interaction with NLRP3. The complex structure was predicted using AlphaFold 3, and key interacting amino acid residues were highlighted using PyMOL. (M) Validation of predicted interaction interface by mutagenesis. Effect of key amino acid mutations on M protein‐LRR domain interaction assessed by co‐IP. M* indicates M protein with key amino acids mutated to glycine; LRR* indicates LRR domain of NLRP3 with key amino acids mutated to glycine. Cell lysates were immunoprecipitated using anti‐Flag antibody and analyzed using anti‐Flag, and anti‐HA antibodies. (N,O) Validation of direct interaction between PDCoV M protein and NLRP3 or its LRR domain by GST pull‐down assay. Purified GST‐tagged M protein (GST‐M) and its mutant form (GST‐M*) were incubated with lysates from HEK‐293T cells transfected with Flag‐NLRP3 (N) or Flag‐LRR domain (O) plasmids for 24 h. Protein complexes were pulled down using glutathione beads and analyzed using anti‐GST and anti‐Flag antibodies. (P) Effect of M protein mutation on NLRP3 inflammasome activation. LLC‐PK cells were transfected with wild‐type M or mutant M* plasmids for 24 h. The NLRP3 inflammasome activation was determined by western blot. Representative results from three independent experiments are shown. Data are expressed as mean ± SD, *n* = 3. Statistical significance was determined using one‐way ANOVA followed by Tukey's multiple comparisons test or two‐way ANOVA followed by Bonferroni's multiple comparisons test. *p < 0.05, **p < 0.01, ***p < 0.001, ****p < 0.0001.

To elucidate how the M protein activates the NLRP3 inflammasome, we examined whether it interacts with inflammasome components (NLRP3, ASC, and pro‐caspase‐1). HEK‐293T cells were co‐transfected with plasmids encoding NLRP3, ASC, and pro‐caspase‐1, together with plasmids encoding PDCoV M. Co‐IP assays revealed that the M protein specifically interacts with NLRP3 but not with ASC or pro‐caspase‐1 (Figure 6A). Endogenous co‐IP in PDCoV‐infected LLC‐PK cells further confirmed this interaction between the M protein and NLRP3 (Figure 6B). To determine the subcellular localization of the M‐NLRP3 interaction, we performed high‐resolution confocal microscopy. In HEK‐293T cells co‐expressing the M protein and inflammasome components (NLRP3 and ASC), the M protein colocalized with NLRP3 spot formation, but not with ASC speck formation (Figure 6C,D). Consistent with these findings, the M protein colocalized with endogenous NLRP3 protein in LLC‐PK cells (Figure 6E,F).

Next, we analyzed the NLRP3 domain critical for M protein binding. Based on its functional domains, NLRP3 was divided into three domains: PYD, NACHT, and LRR (Figure 6I and Table 2). To identify which NLRP3 domain mediates the interaction with the M protein, co‐IP assays demonstrated that the M protein specifically bound to the LRR domain, but not to the NACHT or PYD domains of NLRP3 (Figure 6J). Confocal microscopy further confirmed colocalization of the M protein with the LRR domain in HEK‐293T cells (Figure 6K).

The molecular interface mediating the interaction between NLRP3 and the PDCoV M protein was further assessed using AlphaFold 3‐based three‐dimensional structural modeling to predict the key binding sites of the M–NLRP3 complex. The model revealed that the M protein activates the NLRP3 inflammasome by binding to the LRR domain, which was consistent with the co‐IP and confocal microscopy data (Figure 6L). Subsequently, we analyzed the interacting residues between the LRR domain of NLRP3 and the PDCoV M protein. Structural analysis using the PyMOL revealed that residues M116, H201, T205, K207, R212, Y214, and M217 in the β‐sheet core region (aa 116–217) of the M protein formed hydrogen bond networks with residues Y861, R920, E949, E1007, Y1009, K1015, N1011, and E1033 in the α‐helix groove (aa 742–1037) of the LRR domain (Figure 6L). To validate these predicted critical interaction sites, we generated point‐mutation constructs by substituting all key residues in both the M protein and NLRP3 LRR domain. The mutant plasmids (HA‐M* and Flag‐LRR*) were co‐transfected with their wild‐type counterparts (HA‐M and Flag‐LRR) in HEK‐293T cells, and protein interactions were assessed by co‐IP. The results showed that these mutations abolished the M–LRR interaction (Figure 6M).

To biochemically confirm the direct M–NLRP3 interaction, glutathione S‐transferase (GST) pull‐down assays were performed using purified GST‐M and GST‐M* fusion proteins. GST‐M successfully pulled down full‐length NLRP3 and the LRR domain, demonstrating a direct physical interaction between the M protein and NLRP3 (Figure 6N,O). In contrast, the GST‐M* did not pull down either full‐length NLRP3 or the LRR domain (Figure 6N,O), confirming the specificity of the identified interaction interface. The functional relevance of disrupting this interaction was evaluated by transfecting LLC‐PK cells with the M* mutant construct. Compared with wild‐type M, transfection of M* markedly attenuated caspase‐1 cleavage and IL‐1β maturation (Figure 6P), functionally implicating the M‐LRR interaction in M protein‐driven NLRP3 inflammasome activation. Collectively, these findings identify the LRR domain of NLRP3 as the critical binding interface for the PDCoV M protein and demonstrate that the direct M–NLRP3 interaction at this interface is crucial for M protein‐mediated inflammasome activation and downstream IL‐1β maturation.

### 2.6 PDCoV M Protein Stabilizes NLRP3 by Inhibiting Its Proteasomal Degradation

**FIGURE 7:**
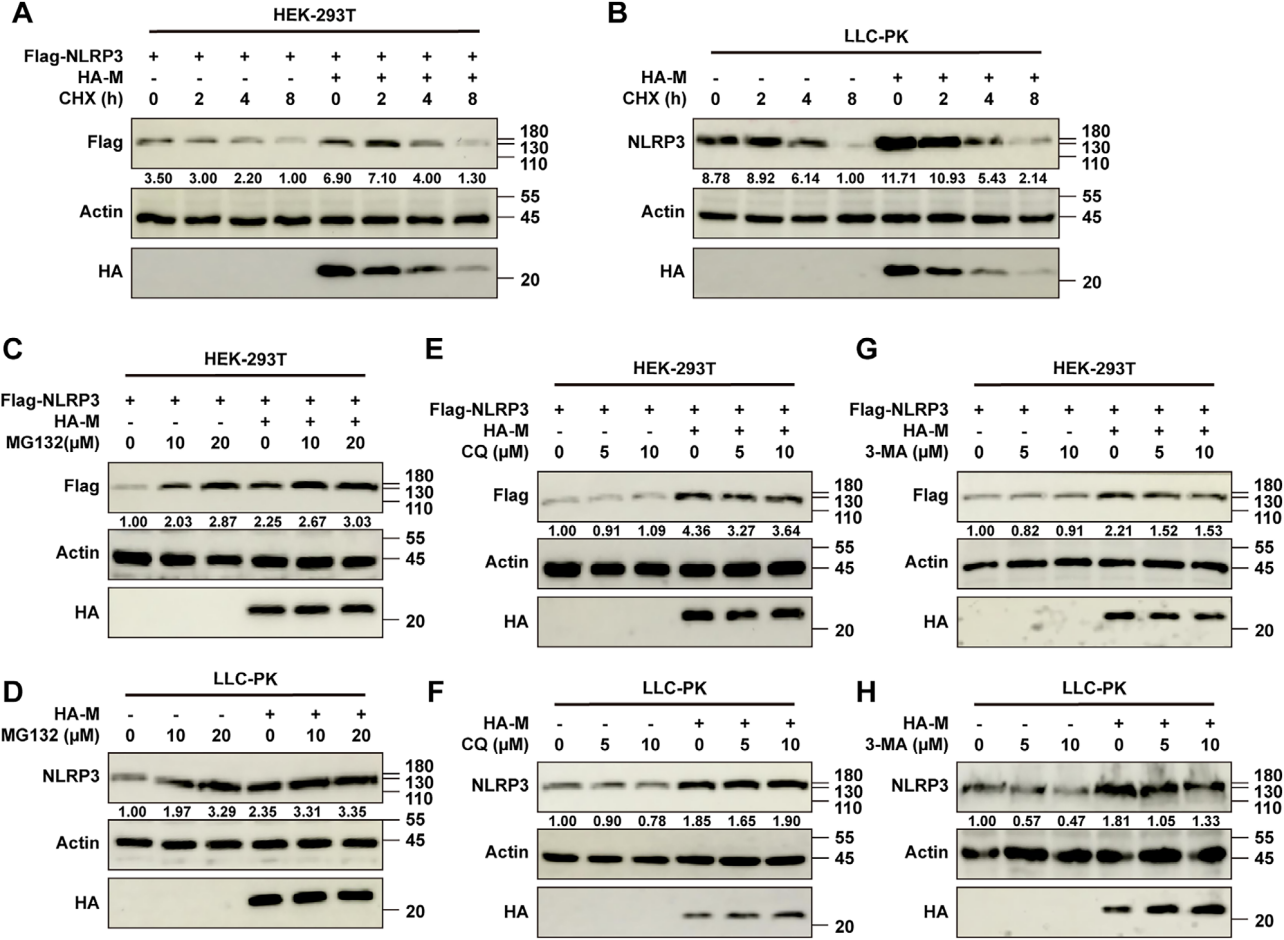
PDCoV M inhibits the proteasomal degradation of NLRP3. (A,B) Effect of the protein synthesis inhibitor cycloheximide on NLRP3 stability. HEK‐293T cells were co‐transfected with Flag‐NLRP3 and HA‐M, and LLC‐PK cells transfected only with HA‐M for 24 h, then treated for various times with cycloheximide (CHX; 100 µg mL^−1^). NLRP3 protein levels determined by western blot. (C–H) Effects of proteasomal and lysosomal inhibitors on NLRP3 stability. HEK‐293T cells were co‐transfected with Flag‐NLRP3 and HA‐M, and LLC‐PK cells transfected only with HA‐M for 24 h, then treated with different doses of MG‐132 (C,D), chloroquine (E,F), and 3‐MA (G,H) for 6 h. NLRP3 protein levels were determined by western blot. Representative results from three independent experiments are shown.

Having established that the M protein promotes NLRP3 inflammasome activation, we next investigated how it upregulates NLRP3 protein expression. To determine whether the M protein affects NLRP3 expression by promoting its synthesis, we treated HEK‐293T cells co‐transfected with Flag‐NLRP3 and HA‐M, as well as LLC‐PK cells transfected with HA‐M, with the protein synthesis inhibitor cycloheximide (CHX). CHX treatment showed that the M protein still promoted the expression of NLRP3 and extended its half‐life compared with the vector control group (Figure 7A,B). This suggests that the M protein stabilizes NLRP3 at the post‐translational level, likely by inhibiting its degradation.

To identify the degradation pathway involved, cells were treated with the proteasome inhibitor MG‐132 at varying concentrations (0, 10, or 20 µM). MG‐132 progressively reduced the difference in NLRP3 levels between M protein‐transfected and vector control cells in both HEK‐293T and LLC‐PK cells (Figure 7C,D), indicating that the M protein stabilizes NLRP3 by inhibiting proteasomal degradation. In contrast, treatment with the lysosome inhibitor chloroquine (CQ) or the autophagy inhibitor 3‐methyladenine (3‐MA) at various concentrations (0, 5, or 10 µM) failed to abolish the M protein‐induced increase in NLRP3 expression; differences between M protein‐transfected and vector control cells persisted under both treatments (Figure 7E–H). Collectively, these results show that the PDCoV M protein stabilizes NLRP3 by selectively inhibiting its proteasomal degradation, rather than its degradation through lysosomal or autophagic pathways.

### 2.7 M Protein Stabilizes NLRP3 by Disrupting TRIM31‐mediated Ubiquitin‐proteasome Degradation

**FIGURE 8:**
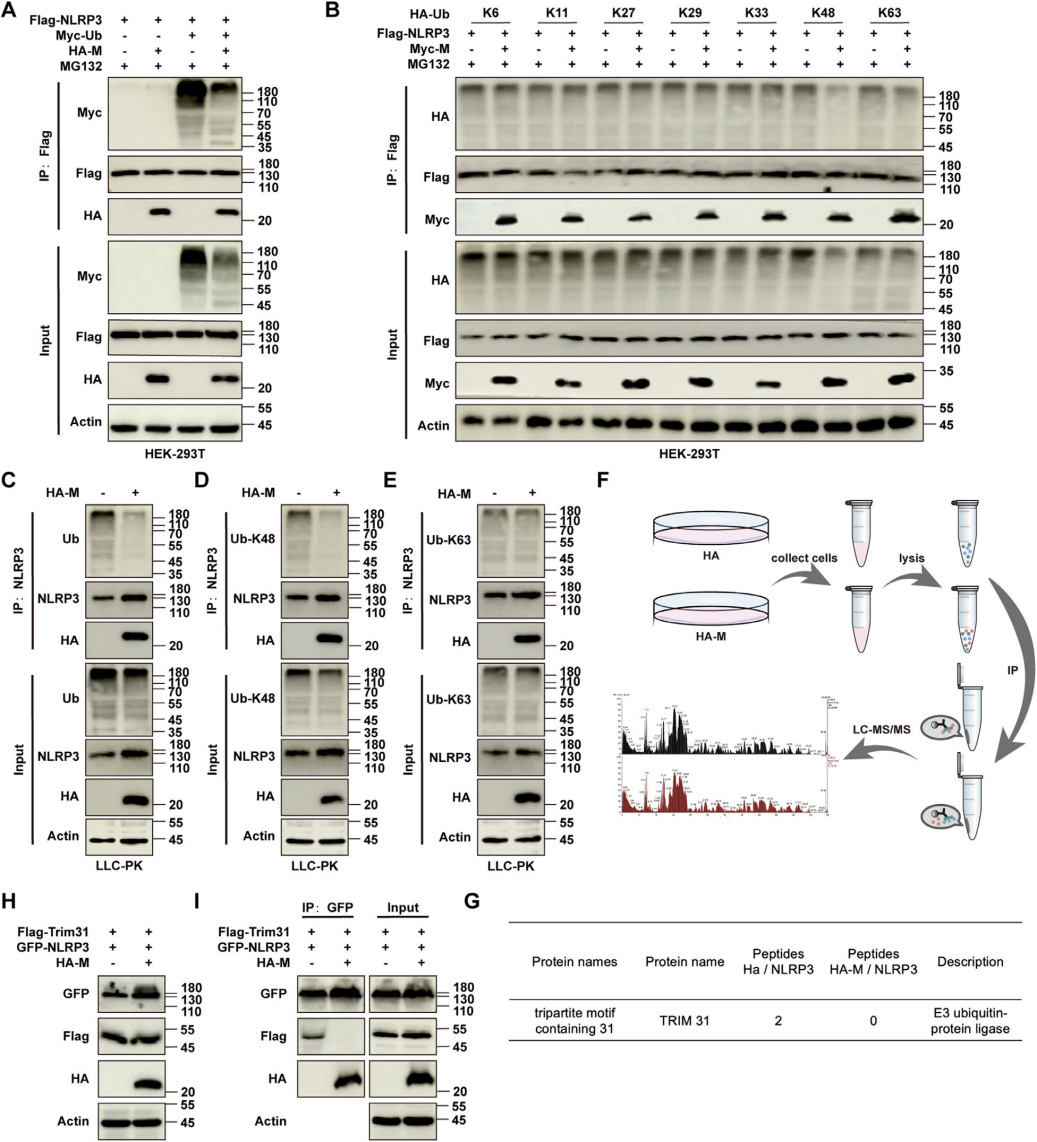
PDCoV M stabilizes NLRP3 by competitively binding to NLRP3 and displacing TRIM31, thereby removing K48‐linked polyubiquitin chains of NLRP3. (A) Effect of PDCoV M protein on NLRP3 ubiquitination. HEK‐293T cells were co‐transfected with Flag‐NLRP3, HA‐M and Myc‐Ub for 24 h, then treated with MG‐132 (20 µM) for 6 h. Cell lysates were immunoprecipitated using anti‐Flag antibody and analyzed using anti‐Flag, anti‐Myc, and anti‐HA antibodies. (B) Identification of ubiquitin linkage types affected by PDCoV M protein. HEK‐293T cells were co‐transfected with Flag‐NLRP3, and HA‐tagged wild‐type ubiquitin or mutants (K6‐, K11‐, K27‐, K29‐, K33‐, K48‐, and K63‐only), together with empty vector (pCMV‐Myc) or Myc‐M for 24 h, then treated with MG‐132 (20 µM) for 6 h. Cell lysates were immunoprecipitated using anti‐Flag antibody and analyzed using anti‐Flag, anti‐Myc, and anti‐HA antibodies. (C–E) Effect of PDCoV M protein on endogenous NLRP3 ubiquitination. LLC‐PK cells were transfected with HA‐M for 24 h, Cell lysates were immunoprecipitated using anti‐NLRP3 antibody and analyzed using anti‐Ub, anti‐Ub‐K48, anti‐Ub‐K63, anti‐NLRP3, and anti‐HA antibodies. (F) Proteins from HEK‐293T cells transfected with vector‐HA or HA‐M and Flag‐NLRP3 were immunoprecipitated using anti‐NLRP3 antibody, and the proteins interacting with NLRP3 were analyzed using LC–MS/MS. (G) LC–MS/MS analysis revealed that NLRP3 recruits the E3 ubiquitin ligase TRIM31, and the M protein interferes with this recruitment. (H) M protein promoted NLRP3 protein expression. HEK‐293T cells were co‐transfected with Flag‐TRIM31, GFP‐NLRP3, and HA‐M for 24 h. NLRP3 protein levels determined by western blot. (I) PDCoV M protein disrupts the TRIM31‐NLRP3 interaction. HEK‐293T cells were co‐transfected with Flag‐TRIM31, GFP‐NLRP3, and HA‐M for 24 h. Cell lysates were immunoprecipitated using anti‐GFP antibody and analyzed using anti‐Flag, anti‐GFP, and anti‐HA antibodies. Representative results from three independent experiments are shown.

Given that the M protein inhibits NLRP3 proteasomal degradation, we next investigated whether it modulates NLRP3 ubiquitination, a prerequisite for proteasomal targeting. HEK‐293T cells were co‐transfected with plasmids encoding Flag‐NLRP3, HA‐M, and Myc‐ubiquitin, and NLRP3 ubiquitination was then assessed by co‐IP. Overexpression of the M protein markedly suppressed total NLRP3 polyubiquitination (Figure 8A). To identify the specific ubiquitin linkage type affected by the M protein, HEK‐293T cells were co‐transfected with plasmids encoding Flag‐NLRP3 and Myc‐M, together with HA‐ubiquitin mutants (K6, K11, K27, K29, K33, K48, and K63), each retaining only lysine for poly‐linkage. Co‐IP revealed that the M protein selectively suppressed K48‐linked polyubiquitination of NLRP3, whereas other linkage types were not effected (Figure 8B). Similarly, LLC‐PK cells were transfected with HA‐M and endogenous NLRP3 ubiquitination was assessed by co‐IP using an anti‐NLRP3 antibody. The results showed that the M protein significantly inhibited ubiquitination of endogenous NLRP3 in LLC‐PK cells, particularly K48‐linked polyubiquitination (Figure 8C–E). Collectively, these results demonstrate that the PDCoV M protein stabilizes NLRP3 by selectively inhibiting K48‐linked polyubiquitination, thereby preventing proteasomal degradation.

To identify the E3 ubiquitin ligase mediating NLRP3 degradation and determine how the M protein interferes, we performed co‐IP coupled with mass spectrometry (co‐IP/MS) in HEK‐293T cells using an anti‐NLRP3 antibody and identified TRIM31, a known E3 ubiquitin ligase, as a candidate NLRP3 interactor (Figure 8F). Compared with the vector control, M protein transfection markedly reduced the association between NLRP3 and TRIM31 (Figure 8G), suggesting that the M protein disrupts TRIM31‐mediated NLRP3 regulation. Co‐expression of GFP‐NLRP3, Flag‐TRIM31, and HA‐M in HEK‐293T cells showed that the M protein promoted NLRP3 protein expression even in the presence of TRIM31 (Figure 8H), consistent with antagonizing of TRIM31‐mediated NLRP3 degradation. Co‐IP further revealed a basal interaction between NLRP3 and TRIM31, which was abolished by the M protein; in contrast, NLRP3 exclusively associated with the M protein (Figure 8I).

In conclusion, these findings demonstrate that the PDCoV M protein competes with TRIM31 for NLRP3 binding, thereby displacing TRIM31 and preventing TRIM31‐mediated K48‐linked polyubiquitination and subsequent proteasomal degradation.

## 3 Discussion

The NLRP3 inflammasome is a critical innate immune sensor that detects viral pathogens and triggers inflammatory responses during CoV infections [54]. Although prior studies have reported NLRP3 activation by various RNA viruses, including SARS‐CoV‐2, PEDV, TGEV, PRRSV, NDV, and IAV [36, 48, 49, 50, 51, 52, 53], the mechanisms underlying PDCoV‐induced NLRP3 inflammasome activation remain unclear. Here, we demonstrate for the first time that PDCoV infection activates the NLRP3 inflammasome both in *vivo* and in *vitro*, promoting IL‐1β maturation and secretion. Most notably, we identified the PDCoV M protein as the key viral factor driving this activation, positioning it as a major proinflammatory factor to PDCoV pathogenesis.

Systematic screening of thirteen viral proteins in reconstituted inflammasome systems provided comprehensive evidence that M protein is the primary PDCoV activator of NLRP3. This finding aligns with reports of coronavirus structural proteins modulating inflammasome but reveals PDCoV‐specific mechanisms. While SARS‐CoV‐2 N proteins regulate inflammasomes [36], our results pinpoint M protein as the dominant PDCoV activator, suggesting species‐specific differences in inflammasome manipulation strategies within the coronavirus family. Notably, M proteins from related porcine coronaviruses PEDV and TGEV failed to activate NLRP3, unlike PDCoV M protein. We speculate that this specificity arises from sequence variability among coronavirus M proteins, highlighting the evolutionary adaptation of PDCoV to exploit host inflammatory pathways [55].

A particularly important finding was the specificity of M protein interaction with the NLRP3 LRR domain, rather than the NACHT or PYD domains, which typically mediate inflammasome oligomerization and downstream signaling. This result suggests that M protein stabilizes NLRP3 in a conformation favorable for complex assembly, rather than directly promoting oligomerization [30, 56]. The LRR domain primarily maintains NLRP3 in an inactive state via intramolecular interactions, making M protein targeting of this domain strategically advantageous for viral pathogenesis [55]. Furthermore, 3D structural modeling and mutagenesis analyses identified specific contacts between M protein residues (M116, H201, T205, K207, R212, Y214, M217) and NLRP3 LRR domain residues (Y861, R920, E949, E1007, Y1009, K1015, N1011, E1033), providing molecular‐level insight into PDCoV M protein‐NLRP3 inflammasome interactions.

The key mechanistic advance is our demonstration that PDCoV M protein competitively displaces TRIM31 from NLRP3, preventing K48‐linked ubiquitination and degradation. TRIM31 acts as a key negative regulator of NLRP3 via its E3 ubiquitin ligase activity, which promotes K48‐linked ubiquitination [57]. The M protein hijacks this pathway by competing for the NLRP3 binding interface, stabilizing the inflammasome and enabling persistent activation. This competitive inhibition represents a novel strategy among viral inflammasome modulators and indicates that PDCoV has evolved sophisticated countermeasures to host negative regulation [58], extending beyond protein interactions to post‐translational interference and underscoring the intricacy of virus‐host interactions.

However, several limitations of our study should be acknowledged. First, we focused primarily on the NLRP3 inflammasome; whether PDCoV M protein affects other inflammasome complexes that contribute to antiviral responses remains unclear [59]. Second, our in *vivo* experiments used 5‐day‐old piglets, and age‐related differences in inflammasome activation and viral susceptibility require further investigation. Third, although we elucidated the M protein–NLRP3 interaction mechanism, the temporal dynamics during natural infection and their relationship to viral replication kinetics need additional characterization.

This study establishes the PDCoV M protein as a critical pro‐inflammatory factor that promotes NLRP3 inflammasome assembly through direct protein‐protein interactions and competitive inhibition of negative regulatory mechanisms. Our findings reveal a sophisticated viral strategy by which PDCoV exploits host inflammatory pathways to facilitate infection while driving disease pathology. The identification of specific M protein‐NLRP3 interaction residues provides molecular targets for therapeutic intervention.

These results extend beyond PDCoV pathogenesis to illuminate coronavirus‐host interactions and viral immune evasion strategies. The competitive displacement of TRIM31 represents a novel paradigm for viral modulation of host regulatory networks, potentially applicable to other coronaviruses or RNA viruses [58, 60]. Viral proteins thus serve dual roles, involving supporting replication while manipulating immunity in pathogen‐beneficial and being detrimental to the pathogen. Future research directions should aim to: 1) evaluate the therapeutic potential of disrupting M protein‐NLRP3 interactions using small molecule inhibitors or peptide competitors; 2) determine the temporal dynamics of inflammasome activation during natural PDCoV infections relative to viral load and disease severity; 3) explore whether M protein–NLRP3 interactions influence broader innate immunity beyond IL‐1β production; and 4) investigate host genetic variations in NLRP3 or TRIM31 affecting PDCoV disease susceptibility. Understanding these mechanisms may yield novel therapeutic strategies for PDCoV and related coronavirus diseases.

## 4 Conclusions

**FIGURE 9:**
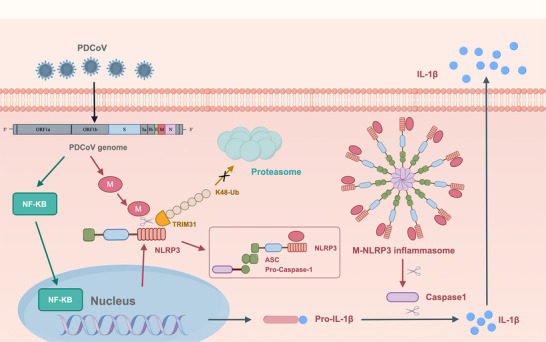
Schematic model by which PDCoV M protein induces NLRP3 inflammasome activation. During PDCoV infection, the membrane (M) protein directly interacts with the LRR domain of NLRP3 protein, thereby promoting the assembly and activation of the NLRP3 inflammasome. Meanwhile, the M protein stabilizes NLRP3 by disrupting TRIM31‐mediated ubiquitin‐proteasome degradation, leading to NLRP3 inflammasome activation and IL‐1β maturation.

In this study, we revealed a novel mechanism by which PDCoV infection activates the NLRP3 inflammasome, thereby driving intestinal inflammation in piglets. Mechanistically, the PDCoV M protein directly interacts with the LRR domain of NLRP3 to promote inflammasome assembly. Notably, the M protein competitively binds NLRP3, displacing the E3 ubiquitin ligase TRIM31 and disrupting TRIM31‐mediated K48‐linked polyubiquitination. This ultimately stabilizes NLRP3 by blocking proteasomal degradation (Figure 9). Together, these findings establish the PDCoV M protein as an essential mediator of NLRP3 inflammasome‐driven inflammatory responses, providing valuable insights into PDCoV pathogenesis that may inform the rational design of antiviral therapeutics or genetically attenuated vaccines for controlling PDCoV infection in piglets.

## 5 Materials and Methods

### 5.1 PDCoV Infection of Piglets

Eight 5‐day‐old piglets were purchased from a commercial pig farm in Henan Province, China. The piglets were randomly divided into mock‐infected (*n* = 4) and PDCoV‐infected groups (*n* = 4). Mock‐infected piglets received 10 mL Minimum Essential Medium Eagle (MEM; Gibco, Thermo Fisher Scientific, Waltham, MA, USA; Cat# 11095080), while each piglet in the PDCoV‐infected group was orally administered 10^8^ TCID_50_ of PDCoV HNZK‐02 strain. Clinical signs were monitored daily. At 3 days post‐infection, when infected piglets showed diarrhea, all animals were euthanized. Serum, intestinal contents, and tissue samples were collected and preserved appropriately.

### 5.2 Ethics Statement

All experimental procedures involving animals were conducted in strict accordance with the ARRIVE guidelines and the guidelines for laboratory animal care and use established by the Ministry of Health, China. The protocol was reviewed and approved by the Animal Care and Ethics Committee of Henan Agricultural University (approval number: HNND2024031408). All necessary precautions were taken to minimize animal suffering throughout the experimental procedures. The study design and implementation adhered to the principles outlined in the Guide for the Care and Use of Laboratory Animals.

### 5.3 Antibodies and Reagents

NLRP3 (Cat# T55651), GFP‐Tag (Cat# M20004), Flag‐Tag (Cat# M20008), Myc‐Tag (Cat# M20002), HA‐Tag (Cat# M20003), Ubiquitin (Cat# T55520), K48‐linkage Specific Ubiquitin (Cat# T55964), K63‐linkage Specific Ubiquitin Antibody (Cat# T56579) were purchased from Abmart (Shanghai, China). Monoclonal mouse anti‐β‐Actin antibody (Cat# HRP‐66009) was obtained from Proteintech (Rosemont, IL, USA). Monoclonal antibodies against Caspase‐1 (Cat# 24232), IL‐1β (Cat# 12703), phospho‐NF‐κB p65(Ser536) (Cat# 3033S) and NF‐κB p65 (Cat# 8242S) were purchased from Cell Signaling Technology (Danvers, MA, USA). HRP‐conjugated Goat Anti‐Mouse IgG (Cat# A0216), HRP‐conjugated Goat Anti‐Rabbit IgG (Cat# A0208), Caspase‐1 Activity Assay Kit (Cat# C1102) were obtained from Beyotime Biotechnology (Shanghai, China). Secondary antibodies for immunofluorescence including Dylight 488 Mouse Anti‐Goat IgG (Cat# A23210), Dylight 594 Mouse Anti‐Goat IgG (Cat# A23420), and Dylight 594 Rabbit Anti‐Goat IgG (Cat# A23421) were purchased from Abbkine (Wuhan, China). Porcine IL‐1β ELISA Kit was obtained from Ray Biotech (Norcross, GA, USA; Cat# ELP‐IL1β). Ac‐YVAD‐cmk (Cat# S8101) was purchased from Selleck Chemicals (Houston, TX, USA). MCC950 (Cat# HY‐12815) and glibenclamide (Cat# HY‐B0513) were obtained from MedChemExpress (MCE; Monmouth Junction, NJ, USA).

### 5.4 Hematoxylin and Eosin (H&E) Staining

Piglet intestinal tissues were isolated and fixed in 10% neutral buffered formalin for 24 h at room temperature, washed with deionized water, dehydrated through a graded ethanol (70%–100%), cleared with xylene, and embedded in paraffin. Tissues were sectioned at a thickness of 4 µm, mounted on slides, and baked at 60°C for 1 h. Sections were dewaxed, rehydrated, stained with hematoxylin and eosin, dehydrated again, cleared, and analyzed under an inverted microscope after mounting with neutral resin.

### 5.5 Cell Lines and Virus

Porcine intestinal epithelial cells (IPEC‐J2), porcine kidney cells (LLC‐PK), and human embryonic kidney cells (HEK‐293T) were obtained from the China Veterinary Drug Control Institute. IPEC‐J2 cells were maintained in DMEM/F12 (Gibco, Thermo Fisher Scientific, Waltham, MA, USA; Cat# 11330032) supplemented with 10% fetal bovine serum (FBS; Vivacell, Shanghai, China; Cat# C04001‐500), whereas LLC‐PK cells were cultured in MEM (Gibco, Thermo Fisher Scientific, Waltham, MA, USA; Cat# 11095080) supplemented with 10% fetal bovine serum (FBS; Gibco, Thermo Fisher Scientific Waltham, MA, USA; Cat# 10437028). HEK‐293T cells were maintained in DMEM (Gibco, Thermo Fisher Scientific Waltham, MA, USA; Cat# 11995065) supplemented with 10% FBS (Vivacell, Shanghai, China; Cat# C04001‐500). All cells were incubated at 37°C with 5% CO_2_.

For virus propagation, IPEC‐J2 and LLC‐PK cells were infected with PDCoV at MOIs of 10, 1, or 0.1. After 1 h of adsorption at 37°C, the inoculum was removed, cells were washed, and maintenance medium containing trypsin at 2.5 mg mL^−1^ for IPEC‐J2 cells and 5.0 mg mL^−1^ for LLC‐PK cells (Gibco, Thermo Fisher Scientific Waltham, MA, USA; Cat# 15090046) was added. Cell lysates and total RNA were collected at 12, 24, and 36 h post‐infection for western blot and qRT‐PCR analysis, respectively.

PDCoV viral titers were determined by TCID_50_ assay. Briefly, IPEC‐J2 cells were seeded in 96‐well plates and infected with 10‐fold serial dilutions of PDCoV. Cytopathic effects (CPE) were observed daily and viral titers were calculated using the Reed‐Muench method.

### 5.6 Plasmids Construction

All PDCoV protein expression plasmids (pCAGGS‐HA‐S, ‐E, ‐M, ‐N, ‐Nsp2, ‐Nsp5, ‐Nsp8, ‐Nsp10, ‐Nsp12, ‐Nsp13, ‐NS6, ‐NS7, and ‐NS7a) were constructed in our laboratory. The porcine NLRP3 and ASC genes were cloned into the pEGFP‐N1 vector to generate GFP‐NLRP3 and GFP‐ASC plasmids. The porcine caspase‐1 gene was cloned into the pCAGGS‐Flag vector to generate Flag‐caspase‐1, while the porcine IL‐1β gene was cloned into the pCMV‐Myc vector to generate Myc‐IL‐1β. Five truncated mutants of porcine NLRP3, designated as Flag‐NLRP3‐D1 (aa 1–217), Flag‐NLRP3‐D2 (aa 1–742), Flag‐NLRP3‐D3 (aa 94–1036), Flag‐NLRP3‐D4 (aa 742–1036), and Flag‐NLRP3‐D5 (aa 217–742) were cloned into the pCAGGS‐Flag vector to generate Flag‐D1, Flag‐D2, Flag‐D3, Flag‐D4, and Flag‐D5 plasmids. The primers used to amplify these genes in this study are listed in Table 1.

HEK‐293T cells were transfected with plasmids using NuLen PlusTrans reagent (Nulen Biotech, Shanghai, China; Cat# CT804) according to the manufacturer's instructions. Cells were incubated for 48 h post‐transfection before further analysis.

### 5.7 Transfections of Cells

LLC‐PK, IPEC‐J2, and HEK‐293T cells were seeded in 12‐well plates containing 1 mL DMEM (Gibco, Thermo Fisher Scientific Waltham, MA, USA; Cat# 11995065) supplemented with 10% FBS (FBS; Vivacell, Shanghai, China; Cat# C04001‐500) without antibiotics. At 80%–90% confluence, cells were transfected with either plasmids or siRNAs. For plasmid transfection, 2.0 µg of expression plasmid was diluted in 100 µL Opti‐MEM (Gibco; Grand Island, NY, USA; Cat# 31985070), while 2.0 µL NuLen PlusTrans reagent (Nulen Biotech, Shanghai, China; Cat# CT804) was diluted separately. For siRNA transfection, 150 nM siRNA and 7.5 µL transfection reagent were diluted individually. After 5 min, solutions were combined and incubated for 20 min at room temperature. Following medium replacement, transfection complexes were added dropwise and incubated for 5 h before changing to complete medium with 2% FBS.

### 5.8 SiRNA Knockdown

RNA interference experiments were performed using synthetic siRNAs targeting NLRP3. The NLRP3 siRNA sequence was as follows: sense, 5'‐GCCUUAAGUUGUGUGAAAUTT‐3'; antisense, 5'‐AUUUCACACAACUUAAGGCTT‐3'. At 48 h post‐transfection, the cells were infected with PDCoV at an MOI of 1.0 for 24 h. The siRNA knockdown efficiency of NLRP3 was assessed by IFA or western blot. IL‐1β levels were measured by western blot or ELISA.

### 5.9 Reconstitution of the NLRP3 Inflammasome in HEK‐293T Cells

HEK‐293T cells were used to reconstitute the NLRP3 inflammasome. When cells reached 60%–80% confluence, they were co‐transfected with 250 ng of GFP‐NLRP3, 250 ng of GFP‐ASC, 500 ng of Flag‐caspase‐1, 500 ng of Myc‐IL‐1β, and 1000 ng of each PDCoV protein expression plasmid (pCAGGS‐HA‐S, ‐E, ‐M, ‐N, ‐Nsp2, ‐Nsp5, ‐Nsp8, ‐Nsp10, ‐Nsp12, ‐Nsp13, ‐NS6, ‐NS7, and ‐NS7a) or empty vector control using NuLen PlusTrans reagent (Nulen Biotech, Shanghai, China; Cat# CT804) according to the manufacturer's instructions. Cells were harvested at 24 h post‐transfection for subsequent analyses.

### 5.10 Enzyme‐Linked Immunosorbent Assay

The concentrations of porcine IL‐1β in cell supernatants, serum, and intestinal contents were measured using enzyme‐linked immunosorbent assay (ELISA) Kit (Norcross, GA, USA; Cat# ELP‐IL1β) according to the manufacturer's instructions. Caspase‐1 activity was determined using caspase‐1 Activity Assay Kit (Beyotime, Shanghai, China; Cat# C1102). For quantification, standard curves were generated using serially diluted standards, and absorbance was measured at 405 nm for caspase‐1 and 450 nm for IL‐1β. All measurements were performed in triplicate.

### 5.11 Western Blot

Cells were lysed with RIPA buffer (Servicebio, Wuhan, China; Cat# G2015‐100ML) containing protease inhibitors. Protein concentrations were determined by the BCA assay (Beyotime, Shanghai, China; Cat# P0010S). Samples were subjected to 8%–12% SDS‐PAGE and transferred to NC membranes (Pall Corporation, Port Washington, NY, USA; Cat# 66485). Membranes were blocked with 5% (w/v) skim milk (Solarbio, Beijing, China; Cat# D8340) in TBST for 2 h, followed by overnight incubation with primary antibodies (1:1000 dilution) at 4°C. After washing, membranes were incubated with HRP‐conjugated secondary antibodies (Beyotime, Shanghai, China; Cat# A0216; 1:12,000 dilution) for 2 h. Protein bands were visualized using enhanced ECL chemiluminescent substrate kit (Yeasen, Shanghai, China; Cat# 36208ES) and images were captured using an Amersham Imager 680 (Cytiva, Little Chalfont, UK; Cat# 29083461).

### 5.12 Co‐Immunoprecipitation (co‐IP)

Piglet intestinal tissues and HEK‐293T cells were washed twice with PBS and lysed with NP40 buffer (Servicebio, Wuhan, China; Cat# G2014). Lysates were centrifuged at 12 000 rpm for 10 min at 4°C. For immunoprecipitation, supernatants were incubated with antibodies bound to Protein A/G magnetic beads (Beyotime, Shanghai, China; Cat# P2179M) overnight at 4°C. Beads were washed three times with TBST. Immunoprecipitated proteins were eluted with 2× SDS‐PAGE sample buffer by boiling for 5 min, followed by centrifugation at 12 000 rpm for 3 min. Proteins were separated by SDS‐PAGE and transferred to NC membranes. Membranes were probed with primary antibodies overnight at 4°C and then with goat anti‐rabbit IgG HRP or mouse anti‐rabbit IgG HRP (avoid light and heavy chain) detection (Abmart, Shanghai, China; Cat# M21004 and M21005; 1:10 000 dilution).

### 5.13 Confocal Microscopy

Cells were fixed with anhydrous ethanol overnight at 4°C and washed three times with PBST. After permeabilization with 0.1% Triton X‐100 for 15 min at room temperature, cells were washed with PBST and blocked with 5% BSA for 30 min at 37°C. Primary antibodies (1:500 dilution) were added and incubated overnight at 4°C. Following three 10 min washes with PBST on a shaker, cells were incubated with secondary antibodies (Dylight 488 Mouse Anti‐Goat IgG, Dylight 594 Mouse Anti‐Goat IgG, or Dylight 594 Rabbit Anti‐Goat IgG; Abbkine, Wuhan, China; Cat# A23210, Cat# A23410 and Cat# A23420; 1:500 dilution) diluted 1:500 in 2% BSA for 2 h at room temperature in the dark. After three 10 min washes with PBST, nuclei were counterstained with DAPI (Servicebio, Wuhan, China; Cat# G1012; 2 µg mL^−1^) for 15 min at room temperature on a shaker. Following three additional 5‐min washes with PBST, cells were mounted with anti‐fade mounting medium (Servicebio, Wuhan, China; Cat# G1401) and stored at 4°C or directly visualized using a confocal laser scanning microscope (ZEISS, Oberkochen, Germany; Model# LSM 900).

### 5.14 Quantitative Real‐Time PCR

Total RNA was extracted from tissues and cells using TRIzol reagent (Vazyme, Nanjing, China; Cat# R401‐01). Tissues and cells were homogenized, incubated with chloroform, and centrifuged at 12 000×g for 10 min. RNA was precipitated with isopropanol, washed with 75% ethanol, and dissolved in DEPC‐treated water. cDNA was synthesized using HiScript II Reverse Transcriptase kit (Vazyme, Nanjing, China; Cat# R223‐01) under conditions: 25°C for 5 min, 50°C for 15 min, and 85°C for 2 min. qPCR was performed using ChamQ SYBR qPCR Master Mix (Vazyme, Nanjing, China; Cat# Q711‐02) in 20 µL reactions containing 10 µL master mix, 1 µL primers, 2 µL cDNA, and 7 µL ddH_2_O. Gene expression was calculated using the 2^−ΔΔCT^ method. Real‐time PCR primers were designed using Primer Premier 5.0 and their sequences were provided in Table S2.

### 5.15 Glutathione S‐Transferase (GST) Pull‐Down

The coding sequences of PDCoV M and its mutant M* were cloned into the pGEX‐4T‐1 and transformed into Escherichia coli strain BL21 (DE3). Protein expression was induced with 0.5 mM IPTG at 16°C for 16 h, and GST, GST‐M, and GST‐M* fusion proteins were purified using glutathione‐Sepharose 4B beads (GE Healthcare). For the pull‐down assay, beads pre‐bound with each fusion protein (5 µg) were incubated with lysates from HEK‐293T cells transfected with Flag‐NLRP3 or Flag‐LRR plasmids for 4 h at 4°C. After washing three times with NP‐40 lysis buffer, bound proteins were eluted in 2× SDS loading buffer, separated by 10% SDS‐PAGE, and analyzed by western blot with anti‐GST and anti‐Flag antibodies.

### 5.16 Statistical Analyses

All statistical analyses were performed using GraphPad Prism 9 software (GraphPad Software, San Diego, CA, USA). The data are shown as mean ± SD, and the sample size (*n*) for each experiment is indicated in the corresponding figure legends. Data were evaluated for normality and homogeneity of variance before ANOVA analysis. Statistical significance was determined using one‐way ANOVA followed by Tukey's multiple comparisons test or two‐way ANOVA followed by Bonferroni's multiple comparisons test, as indicated in each figure legend. All statistical tests were two‐sided. *p* < 0.05 was considered statistically significant. *p < 0.05; **p < 0.01; ***p < 0.001; ****p < 0.0001; ns, not significant.

## Author Contributions

**Jinhui Hou**: Writing – original draft, Methodology, Investigation, Formal analysis, Data curation. **Fangfang Han**: Methodology, Investigation, Data curation, Formal analysis. **Anqi Liu**: Methodology, Investigation, Data curation, Formal analysis. **Yang Yu**: Methodology, Investigation, Data curation, Formal analysis. **Rui Zhao**: Methodology, Investigation. **Zhengting Shi**: Methodology, Investigation. **Yanrong Lv**: Resources, Validation, Visualization. **Jinli Liu**: Resources, Formal analysis. **Shaopo Zu** and **Jin Yuan**: Writing – original draft, Writing – review & editing, Supervision, Funding acquisition, Project administration, Conceptualization. **Zhanyong Wei** and **Hui Hu**: Writing – review & editing, Supervision, Project administration, Funding acquisition, Conceptualization.

## Funding

The National Key Research and Development Program of China (2023YFD1801800). The National Natural Science Foundation of China (32130106, 32402924, and U22A20520). Science and Technology Innovation Leading Talent Support Program of Henan Province (254200510033). Program for Innovative Research Team (in Science and Technology) in University of Henan Province (24IRTSTHN034). Outstanding Young Innovation Research Group Project of Natural Science Foundation of Henan Province (262300421002).

## Ethics Approval

Animal experiments were approved by the Animal Care and Ethics Committee of Henan Agricultural University (approval number: HNND2024031408). The study was conducted in strict accordance with the ARRIVE guidelines and the guidelines for the care and use of laboratory animals established by the Ministry of Health, China. All necessary measures were taken to minimize animal suffering throughout the experimental procedures.

## Conflicts of Interest

The authors declare no conflicts of interest.

## Supporting information




**Supporting File 1**: advs76393‐sup‐0001‐SuppMat.docx.

**Supporting File 2**: advs76393‐sup‐0002‐FigureS1‐S3.zip.

## Data Availability

The data that supports the findings of this study are available in the supplementary material of this article.
