# Supplementary material for: Porcine Deltacoronavirus M Protein Binds NLRP3 to Promote Inflammasome Assembly via Competition with TRIM31
Source: Adv Sci (Weinh). 2026 Jun 30;13(54):e76393. doi: 10.1002/advs.76393 (PMC13336972; doi:10.1002/advs.76393)
Supplement: Supplementary file 1 [file ADVS-13-e76393-s002.docx]

Supporting Information

Porcine Deltacoronavirus M Protein Binds NLRP3 to Promote Inflammasome Assembly via Competition with TRIM31

*Jinhui Hou#, Fangfang Han#, Anqi Liu#, Yang Yu, Rui zhao, Zhengting Shi, Yanrong Lv, Jinli Liu, Shaopo Zu, Zhanyong Wei, Jin Yuan*, Hui Hu**

**Supplement figure 1**

**Supplement figure 1. PDCoV infection induces inflammatory responses in *vivo*. Related to Figure 1.**

(A-G) The critical inflammatory factors mRNA levels of in serum and intestinal contents at 3 dpi determined by qRT-PCR. IL-6(A), IL-8(B), TNF-a(C), IFN-a(D), ccl2(E), ccl20(F), cxcl2(G).

(H) p65 and p-p65 in the small intestine at 3 dpi determined by western blot. Lanes 1–3: PDCoV-infected piglets; Lanes 4-6: mock-infected control piglets. Representative results from three independent experiments are shown. Data are representative of three independent experiments. Data are expressed as mean ± SD, n = 3. Statistical significance was determined using one-way ANOVA followed by Tukey’s multiple comparisons test or two-way ANOVA followed by Bonferroni’s multiple comparisons test. **P* < 0.05, ***P* < 0.01, ****P* < 0.001.

**Supplement figure 2**

**Supplement figure 2. PDCoV infection induces ASC oligomerization and caspase-1 cleavage *in vitro.*** **Related to Figure 2.**

(A-B) IPEC-J2 (A) and LLC-PK (B) cells infected with PDCoV at different MOI (10, 1, 0.1) for 24 h. cells were lysed and the pellets were subjected into cross-link. ASC oligomerization in the pellets and the total ASC in lysates as the input were determined by western blot.

(C-D) IPEC-J2 (C) and LLC-PK (D) cells infected with PDCoV (MOI = 1) at different time points (12, 24, 36 h), cells were lysed and the pellets were subjected into cross-link. ASC oligomerization in the pellets and the total ASC in lysates as the input were determined by western blot.

(E-F) IPEC-J2 (E) and LLC-PK (F) cells infected with PDCoV (MOI = 1) at different time points (12, 24, 36 h). Caspase-1 enzyme activity in cell lysates was measured by ELISA.

(G-H) IPEC-J2 (E) and LLC-PK (F) cells infected with PDCoV at different MOI (10, 1, 0.1) for 24 h. Caspase-1 enzyme activity in cell lysates was measured by ELISA.

(I-L) IPEC-J2 (I, K) and LLC-PK (J, L) cells infected with PDCoV (MOI = 1) at different time points (12, 24, 36 h) (I-J) and infected with PDCoV at different MOI (10, 1, 0.1) for 24 h (K-L). LDH release in culture supernatant was measured by ELISA.

(M-N) IPEC-J2 (M) and LLC-PK (N) cells treated with AC-YVAD-CMK at different concentrations (0.1, 1, 10 μM) for 3 h, then infected with PDCoV at an MOI =1 for 36 h. LDH release in culture supernatant was measured by ELISA.

Representative results from three independent experiments are shown. Data are expressed as mean ± SD, n = 3. Statistical significance was determined using one-way ANOVA followed by Tukey’s multiple comparisons test or two-way ANOVA followed by Bonferroni’s multiple comparisons test. **P* < 0.05, ***P* < 0.01, ****P* < 0.001, *****P* < 0.0001.

**Supplement figure 3**

**Supplement figure 3. PDCoV infection induces IL-1β maturation and secretion through the activation of NLRP3 inflammasomes in *vitro.*** **Related to Figure 4.**

(A) PDCoV-induced NLRP3 inflammasome assembly in IPEC-J2 and LLC-PK cells detected by co-IP. Cells were infected with PDCoV (MOI = 1) for 36 h or mock-infected. Cell lysates were immunoprecipitated using anti-ASC antibody or control IgG, then analyzed using anti-NLRP3, anti-ASC and anti-pro-caspase-1 antibodies.

(B-C) Effect of NLRP3 knockdown on PDCoV-induced inflammasome activation. IPEC-J2 and LLC-PK cells were transfected with siNLRP3, or siNC (negative control) at 150 nM for 24 h, then infected with PDCoV (MOI = 1) for 36 h. LDH release in culture supernatant was measured by ELISA.

(D-G) Effect of NLRP3 inflammasome inhibitors on PDCoV-induced inflammasome activation. IPEC-J2 and LLC-PK cells were pretreated with MCC950 (D-E) or glibenclamide (F-G) at different concentrations (0.1, 1, 10 μM) for 3 h, then infected with PDCoV (MOI = 1) for 36 h. LDH release in culture supernatant was measured by ELISA.

Representative results from three independent experiments are shown. Data are expressed as mean ± SD, n = 3. Statistical significance was determined using one-way ANOVA followed by Tukey’s multiple comparisons test or two-way ANOVA followed by Bonferroni’s multiple comparisons test. **P* < 0.05, ***P* < 0.01, ****P* < 0.001, ****P < 0.0001.
