# Supplementary figures and images for: Porcine Deltacoronavirus M Protein Binds NLRP3 to Promote Inflammasome Assembly via Competition with TRIM31
Source: Adv Sci (Weinh). 2026 Jun 30:e76393. Online ahead of print. doi: 10.1002/advs.76393 (PMC13336972; doi:10.1002/advs.76393)

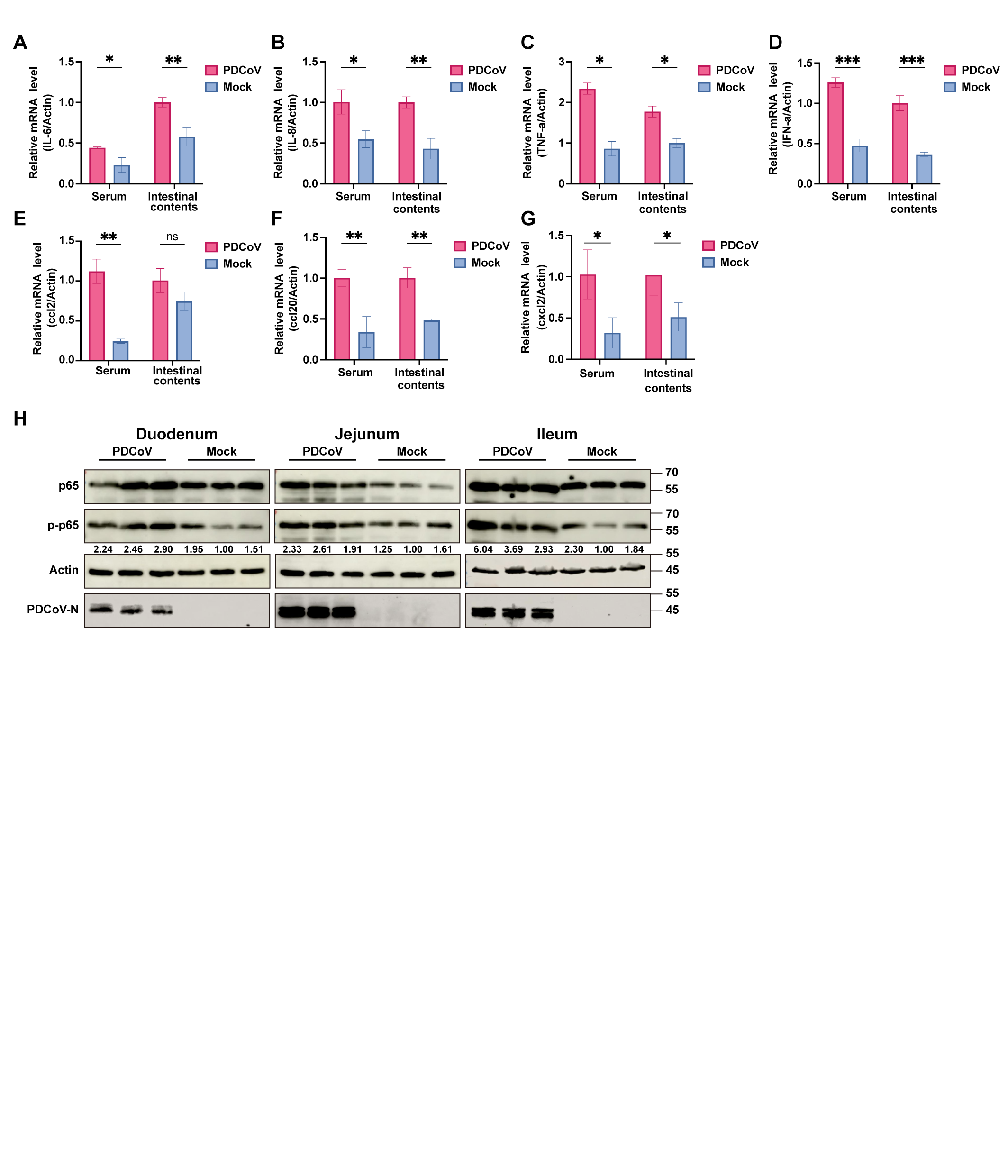

Supplement: Supplementary file 2 — Supporting File 2: advs76393‐sup‐0002‐FigureS1‐S3.zip. [file ADVS-9999-e76393-s002.zip › Sup Fig 1.tif]

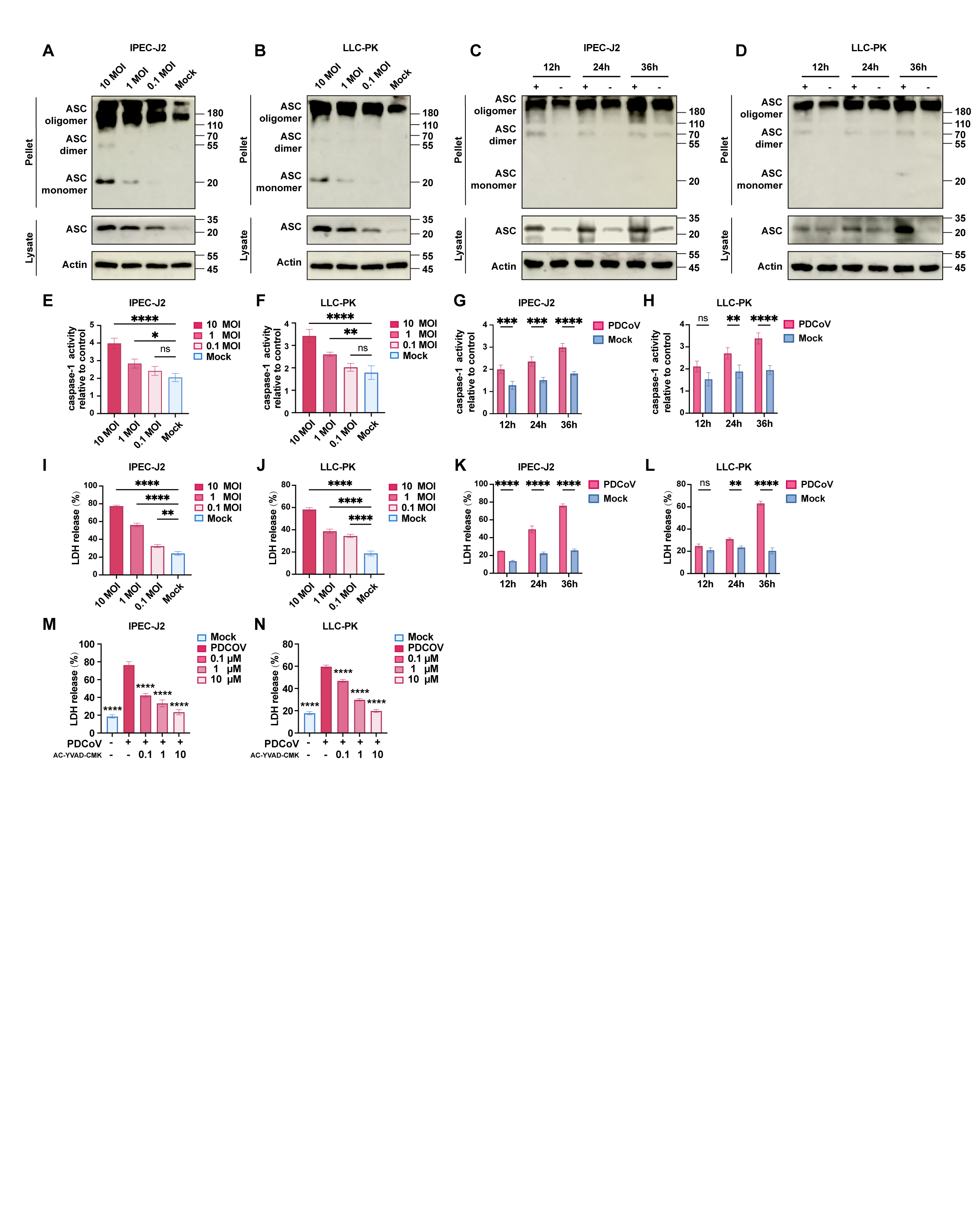

Supplement: Supplementary file 2 — Supporting File 2: advs76393‐sup‐0002‐FigureS1‐S3.zip. [file ADVS-9999-e76393-s002.zip › Sup Fig 2.tif]

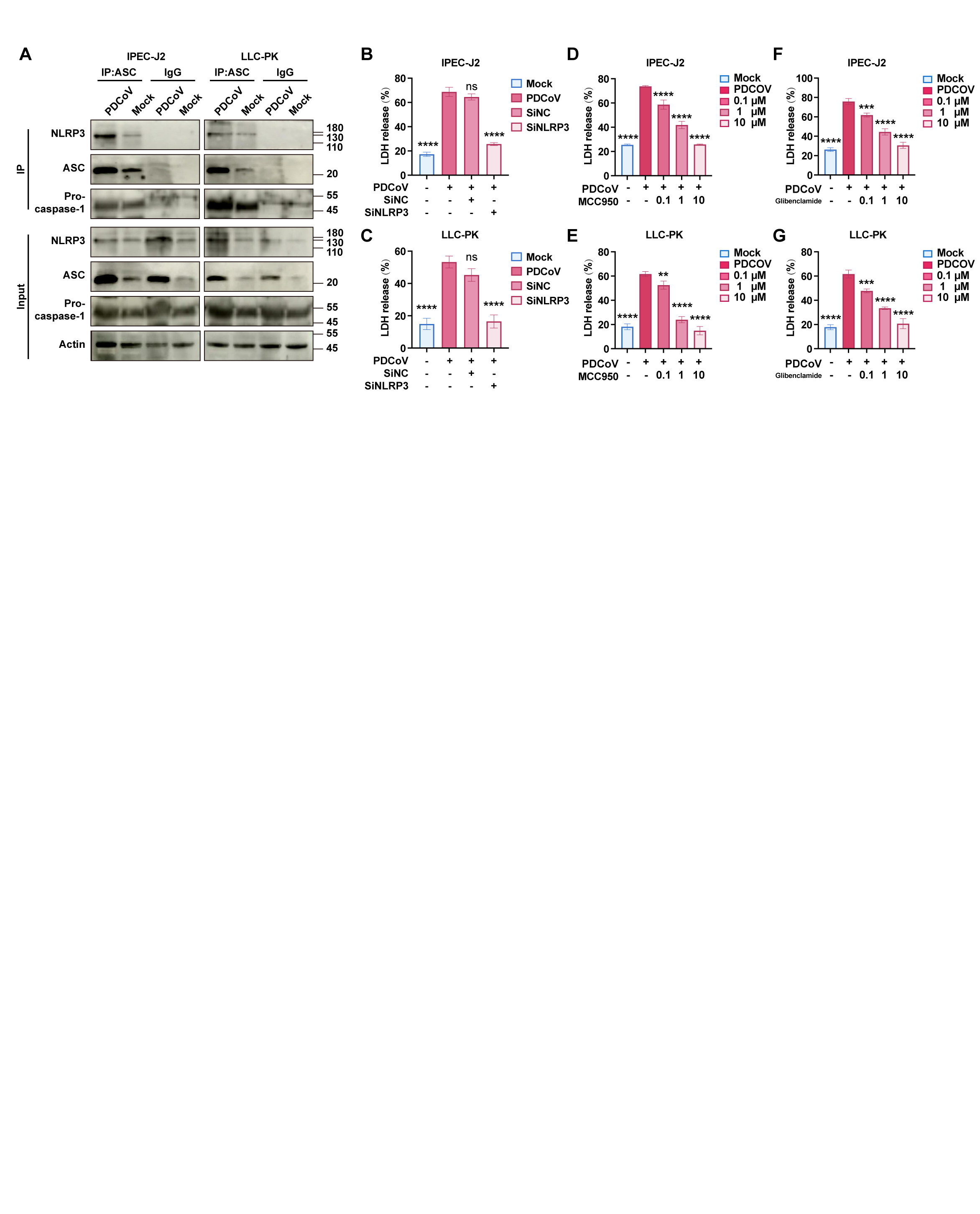

Supplement: Supplementary file 2 — Supporting File 2: advs76393‐sup‐0002‐FigureS1‐S3.zip. [file ADVS-9999-e76393-s002.zip › Sup Fig 3.tif]
